# Real‐Time Evolutionary Landscape of the Bronchial Epithelium and Corresponding Dynamic Immune Cell Alterations in Lung Squamous Cell Carcinogenesis

**DOI:** 10.1002/advs.202413256

**Published:** 2025-06-05

**Authors:** Baohong Luo, Yuting Luo, Sicheng Chen, Tiantian Yang, Bixia Liu, Xiting Liao, Xiaoke Zheng, Tian Tian, Jinxu Liu, Qinru Zhan, Xiaohua Situ, Zhongpeng Xie, Yanxia Wang, Zhe‐Sheng Chen, Honglei Chen, Zheng Yang, Zunfu Ke

**Affiliations:** ^1^ Department of Pathology The First Affiliated Hospital Sun Yat‐sen University Guangzhou Guangdong 510000 P. R. China; ^2^ Molecular Diagnosis and Gene Test Center The First Affiliated Hospital Sun Yat‐Sen University Guangzhou Guangdong 510000 P. R. China; ^3^ Department of Pathology The Seventh Affiliated Hospital Sun Yat‐sen University Shenzhen Guangdong 518000 P. R. China; ^4^ Department of Pharmaceutical Sciences College of Pharmacy and Health Sciences St. John's University Queens, New York 11439 USA; ^5^ Department of Pathology Zhongnan Hospital Wuhan University Wuhan Hubei 430000 P. R. China

**Keywords:** carcinogenesis, immune microenvironment, lung squamous cell carcinoma, premalignant lesions, single‐cell RNA sequencing

## Abstract

The molecular mechanism by which tumor cells and their microenvironment evolve during lung squamous cell carcinoma (LUSC) carcinogenesis remains unclear, greatly limiting its early diagnosis and treatment effectiveness in patients. To replicate pathogenic processes and identify the determinants of cell evolution, a rat model is established using tobacco‐derived carcinogens. Here, a series of single‐cell transcriptome profiles of normal lung epithelium, hyperplasia, metaplasia, dysplasia, and squamous cell carcinoma in situ (CIS) to invasive squamous cell carcinoma (SCC) is presented. A large proportion of canonical copy number variations (CNVs) are detected in the hyperplasia/metaplasia stages, with their frequency increasing as the lesion progressed. Although bronchial epithelial cells exhibit substantial heterogeneity, three distinct cellular states are identified during their evolution into malignant cells. Immune sensing occurs at the earliest stages of cellular transformation. However, persistent exposure to carcinogens induces microenvironmental remodeling, which is characterized by monocyte‐derived macrophage infiltration, plasmacytoid dendritic cell expansion, and progressive T‐cell exhaustion. These findings depict the evolutionary trajectory of cancer and the immune microenvironment, emphasizing the need for CNV evaluation in early screening and immune‐based therapy for lesions at a high risk of progression to LUSC.

## Graphical abstract

Lung squamous cell carcinoma (LUSC) is often preceded by sequential premalignant lesions with an increasing probability of malignant progression. Using single‐cell RNA sequencing, the dynamic cellular evolution from normal lung to LUSC tissues is delineated; clonal structure of premalignant and malignant epithelium is reveled; the conversion of immune sensing to immunosuppression during lung squamous carcinogenesis is characterized.

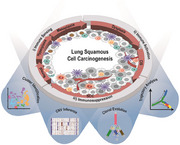

## 1 Introduction

Lung squamous cell carcinoma (LUSC) remains the second most common subtype of nonsmall cell lung cancer, accounting for ≈20–30% of lung cancer cases worldwide. No first‐line targeted agents have been approved for the treatment of this disease^[^ ^1^, ^2^ ^]^ Although there are recurrent molecular aberrations in LUSCs, including mutations in EGFR, FGFR, and PIK3, and loss of the CDKN2A tumor suppressor, therapeutic regimens targeting these molecules have largely failed.^[^ ^3^ ^]^ Immune checkpoint inhibitors that block the PD‐1/PD‐L1 interaction have demonstrated durable tumor regression; however, the response rate is unsatisfactory.^[^ ^4^, ^5^ ^]^ The limited clinical efficacy of current therapeutic agents underscores the incomplete understanding of molecular targets (e.g., genomic alterations) in LUSC, highlighting the necessity to reveal the evolutionary features of tumor cells and the microenvironment during LUSC carcinogenesis.

LUSC is a malignant epithelial tumor originating in the bronchial mucosa.^[^ ^6^ ^]^ Invasive LUSC is often preceded by sequential premalignant stages, including hyperplasia, metaplasia, dysplasia, and squamous cell carcinoma in situ (CIS), with an increasing probability of malignant progression.^[^ ^7^, ^8^ ^]^ Although dysplastic lesions rarely progress to invasive squamous cell carcinoma (SCC), persistent dysplasia, especially high‐grade lesions, significantly increase the risk of developing SCC.^[^ ^9^, ^10^ ^]^ Researchers have attempted to identify transcriptomic alterations during different stages of LUSC carcinogenesis using mRNA sequencing or gene expression profiling.^[^ ^11^, ^12^, ^13^, ^14^ ^]^ However, owing to the heterogeneity of human samples and the rarity of preinvasive lesion collections, the molecular events underlying the carcinogenic process remain elusive. To overcome these challenges, preclinical animal models have been developed that provide valuable insights into aspects of LUSC biology.^[^ ^15^ ^]^ However, these models do not accurately represent the stepwise accumulation of genetic alterations from normal tissue to invasive carcinoma. Thus, a better model to depict the successive stages of carcinogenesis and elucidate tumor cell evolutionary trajectories at different time points, as well as dynamic alterations in the immune microenvironment, is urgently needed for LUSC research.

In this study, we combined single‐cell RNA sequencing (scRNA‐seq) and integrated analysis to perform comprehensive single‐cell transcriptome profiling from normal lung tissues to successive lesion tissues during LUSC carcinogenesis. An LUSC model was induced in Wistar rats using the carcinogens 3‐methylcholanthrene (3‐MCA) and diethylnitrosamine (DEN), which are similar to human LUSC.^[^ ^16^, ^17^, ^18^ ^]^ By deciphering the molecular landscape of carcinogenesis, we offer new insights into the dynamic molecular co‐evolution of bronchial and immune cells in lung tissues during different morphological stages of LUSC.

## 2 Results

### 2.1 Dynamic Cellular Evolution from Normal Lung Epithelium to LUSC

**Figure 1:**
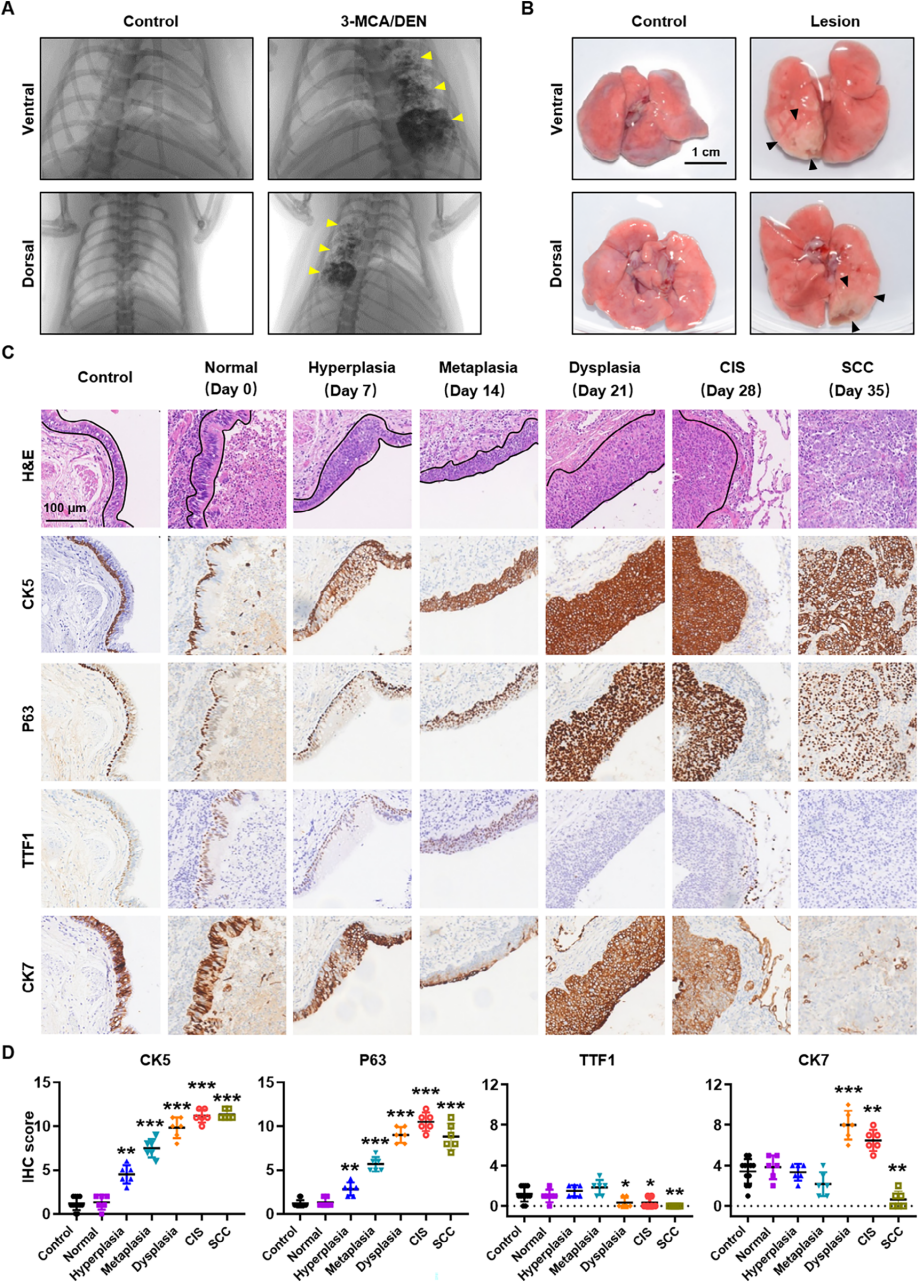
Photomicrographs of typical LUSC development in MCA/DEN‐induced Wistar rats. A, Wistar rats were intratracheally instilled with 100 µl of iodized oil containing 10 mg of 3‐MCA and 10 µL of DEN, followed by imaging detection via an animal micro‐CT scan. B, Representative gross images of normal lung tissue and pulmonary lesions. The black arrows point to visible lesions. Scale bar, 1 cm. C, Representative H&E and IHC images of different lesion stages ranging from normal tissue to hyperplasia, metaplasia, dysplasia, CIS, and SCC (paraffin‐embedded tissue sections). Scale bar, 100 µm. D, The IHC scores of CK5, P63, TTF1, and CK7 for all samples. *p < 0.05, **p < 0.01, ***p < 0.001; one‐way ANOVA with Tukey's post hoc test was used. 3‐MCA, 3‐methylcholanthrene; DEN, diethylnitrosamine; H&E, hematoxylin and eosin; IHC, immunohistochemistry; CIS, carcinoma in situ; SCC, squamous cell carcinoma; CK5, cytokeratin 5; TTF1, thyroid transcription factor 1; CK7, cytokeratin 7.

The LUSC model was established in Wistar rats with monitoring via micro‐CT (**Figure** **1**A). The lung tissues were collected at predetermined intervals after carcinogen instillation (Figure 1B). Comprehensive histopathological assessments, including hematoxylin and eosin (H&E) staining and immunohistochemistry (IHC), were performed to characterize the temporal progression of bronchial epithelial lesions. This multistep carcinogenic process was histologically confirmed to encompass the full spectrum of epithelial transformation, ranging from normal bronchial epithelium through sequential stages of hyperplasia, metaplasia, dysplasia, and CIS, to invasive SCC (Figure 1C,D, Figure S1, Supporting Information).

**Figure 2:**
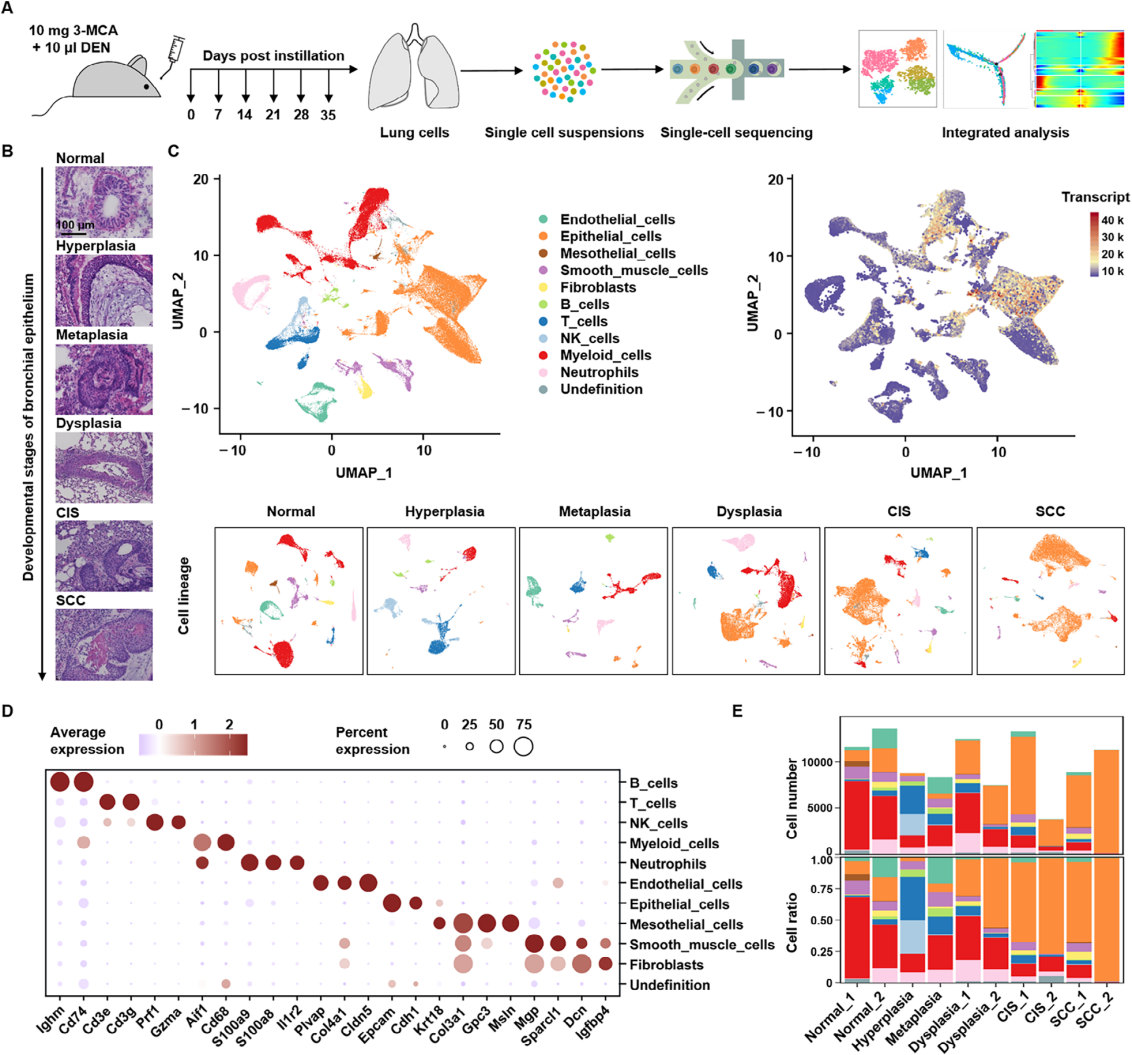
Single‐cell transcriptomic analysis revealing the cellular dynamic changes during lung squamous cell carcinogenesis. A, Schematic illustration of the study design. Wistar rats were intratracheally instilled with 10 mg of 3‐MCA and 10 µl of DEN, followed by sampling on days 0, 7, 14, 21, 28, and 35. Single‐cell RNA sequencing analysis with 10x Genomics was performed on lung tissues at stages of normal epithelium, hyperplasia, metaplasia, dysplasia, CIS, and SCC. B, Representative morphological stages of LUSC development (fresh frozen tissue sections). Scale bar, 100 µm. C, UMAP plot showing integrated lung cells from 10 rats across different stages of lung squamous cell carcinogenesis. Left: 11 clusters labeled by major cell types. Right: cells colored by transcript count. Bottom: UMAP plots for each stage of carcinogenesis. D, Dot plot displaying the average expression of canonical marker genes for the 11 major lineages depicted in (C). The area of the dots represents the proportion of cells expressing these marker genes, and the color intensity of the dots corresponds to the gene expression level. E, Stacked bar plot showing the relative proportion of each cell cluster across 10 samples as indicated.

Droplet‐based scRNA‐seq was performed on 99277 single cells from lung tissues at different stages of LUSC carcinogenesis, representing all gene expression profile subtypes and annotated cell types (**Figure** **2**A–C, Table S1, Supporting Information). The number of unique molecular indices (UMIs) detected per cell ranged from 1063 to 8170, and the average gene count varied between 629 and 2372 (Figure S2, Supporting Information). Unbiased clustering in Seurat identified ten cell subclusters as common cell types expressing distinct canonical markers, which were derived from epithelial cells (including bronchial, alveolar, and cancer cells), stromal cells (including fibroblasts, mesothelial cells, endothelial cells, and smooth muscle cells), and immune cells (including T cells, B cells, natural killer [NK] cells, neutrophils, and myeloid cells) (Figure 2D, Figures S3–S4, Tables S2, Supporting Information).

In addition to normal lung tissues, all cell subtypes were observed in the lesion tissue at each stage, and their composition ratio varied greatly (Figure 2E, Figures S5–S6, Supporting Information). Considering the multiple disease statuses and limited number of samples, we also used the sccomp framework for differential composition and variability analysis.^[^ ^19^ ^]^ A linear increase in epithelial cell proliferation occurred predominantly after dysplasia. In contrast, T cell, B cell, and NK cell enrichment was mainly observed before dysplasia, indicating that immune sensing or response could be activated in the early stages of cancer. However, once cancer lesions form, especially in the invasive stage, the number of immune cells, such as T, B, and NK cells, decreases rapidly, reflecting an immunosuppressive state. Additionally, a reduction in myeloid‐derived cells was detected in tumor lesions, including CIS and SCC (Figure 2E, Figure S7, Supporting Information). These data suggest that dynamic changes in the epithelial and immune cells play vital roles in LUSC development.

### 2.2 Single‐Cell Copy Number Variation (CNV) Analysis Probes the Clonal Structure of Premalignant and Malignant Epithelium

Although 3‐MCA and DEN promote lung cancer development,^[^ ^16^, ^17^, ^18^ ^]^ the intrinsic characteristics of genetic alterations following exposure to these genotoxic carcinogens remain unclear. In this study, after two consecutive rounds of unsupervised clustering, epithelial cells were classified as basal/tumor cells, ciliated cells, alveolar type II (AT2) cells, alveolar type I (AT1) cells, brush cells, and club cells in both normal lung and lesion lung tissues (Figure S8A–C, Supporting Information). As shown in Figure S8D (Supporting Information), brush cells were completely absent after dysplasia (Figure S8D, Supporting Information). Conversely, the proportion of basal/tumor cells gradually increased with lesion progression (Figure S8D, Supporting Information), consistent with the stepwise morphological evolution observed via H&E staining, supporting the classic view that LUSC originates from preinvasive progenitor cells in the central airways.^[^ ^7^ ^]^

**Figure 3:**
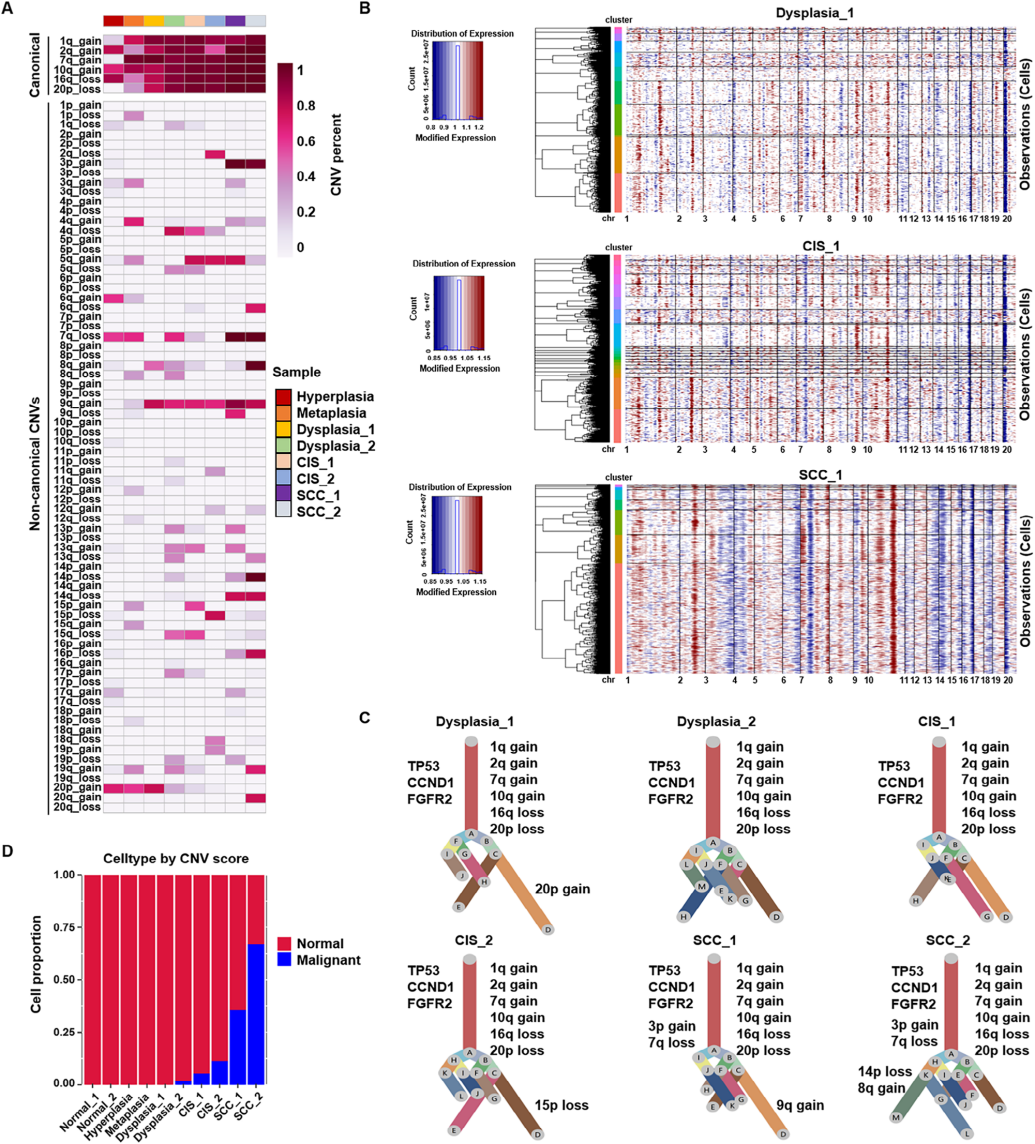
Single‐cell CNV analysis probing the clonal evolution of epithelial cells. A, Summary CNV profiles of single epithelial cells from each lesion. The CNV levels were annotated based on their location within chromosomes, in which the CNV events calculated by inferCNV were simplified as either gain or loss. The color scale in the heatmap indicates the percentage of CNV events. B, Representative hierarchical heatmaps from the inferCNV algorithm reflecting the distribution of CNVs in the epithelium from a dysplasia lesion, a CIS lesion and an SCC lesion (Figure 10SA for the other 5 lesions in the Supporting Information). The annotation track on the left indicates the epithelial cell subclusters depicted in Figure S9 in the Supporting Information. The red blocks and blue blocks represent copy number gains and copy number losses, representatively. C, Clonal evolutionary trees of dysplasia, CIS, and SCC lesions generated via uphyloplot2 mapping with CNVs and relevant key genes. The branches were delineated according to the percentage of cells in the subclone containing the corresponding CNVs. The annotation revealed the canonical CNVs in each sample (Figure S10B in the Supporting Information for the other two lesions). D, Proportion of malignant cells in each stage during the carcinogenic process of LUSC, inferred from the CNVs in the branch of the clonality tree indicated in (**C**).

To distinguish tumor cells from basal cells, we used the inferCNV algorithm to obtain large‐scale CNVs by averaging the relative RNA levels over large genomic regions.^[^ ^20^ ^]^ The annotated epithelial cells from each sample were subjected to unsupervised subclustering, followed by CNV inference (Figure S9, Supporting Information). The complexity of the CNVs is displayed across the chromosomal landscape. While canonical CNVs dominated the chromosome arm (>90% of cells) and increased with lesion progression, there were multiple subclonal noncanonical CNVs across the lesions (**Figure** **3**A,B, Figure S10A, Supporting Information). Based on the CNV analysis, the UPhyloplot2 plotting algorithm was used to generate a clonality tree for lung lesions.^[^ ^21^, ^22^ ^]^ Prominent canonical chromosomal variations consisted of gains in 1q, 2q, 7q, and 10q as well as losses in 16q and 20p, which were associated with key genes, including TP53, CCND1, and FGFR2 (Figure 3C, Figures S10B and S11, Supporting Information). Most canonical CNV subclones in dysplasia persisted in the CIS and SCC lesions, suggesting that the continued existence of canonical CNVs plays an important role in promoting genomic evolution (Figure 3C). According to the CNV score, malignant cells exhibited remarkably higher CNV levels than the normal epithelial cells (Figure S8E,F, Supporting Information). Furthermore, the copy number profiles of malignant cells inferred from scRNA‐seq data were generally similar to those obtained from whole‐genome sequencing (Figure S12, Supporting Information). Interestingly, cells presumed to be malignant first appeared in the dysplastic stage rather than in the CIS stage (Figure 3D), indicating the occurrence of irreversible molecular and cellular events in the precursor stage of tumor development.

### 2.3 Single‐Cell Trajectory Branches Predict Cell Evolution

**Figure 4:**
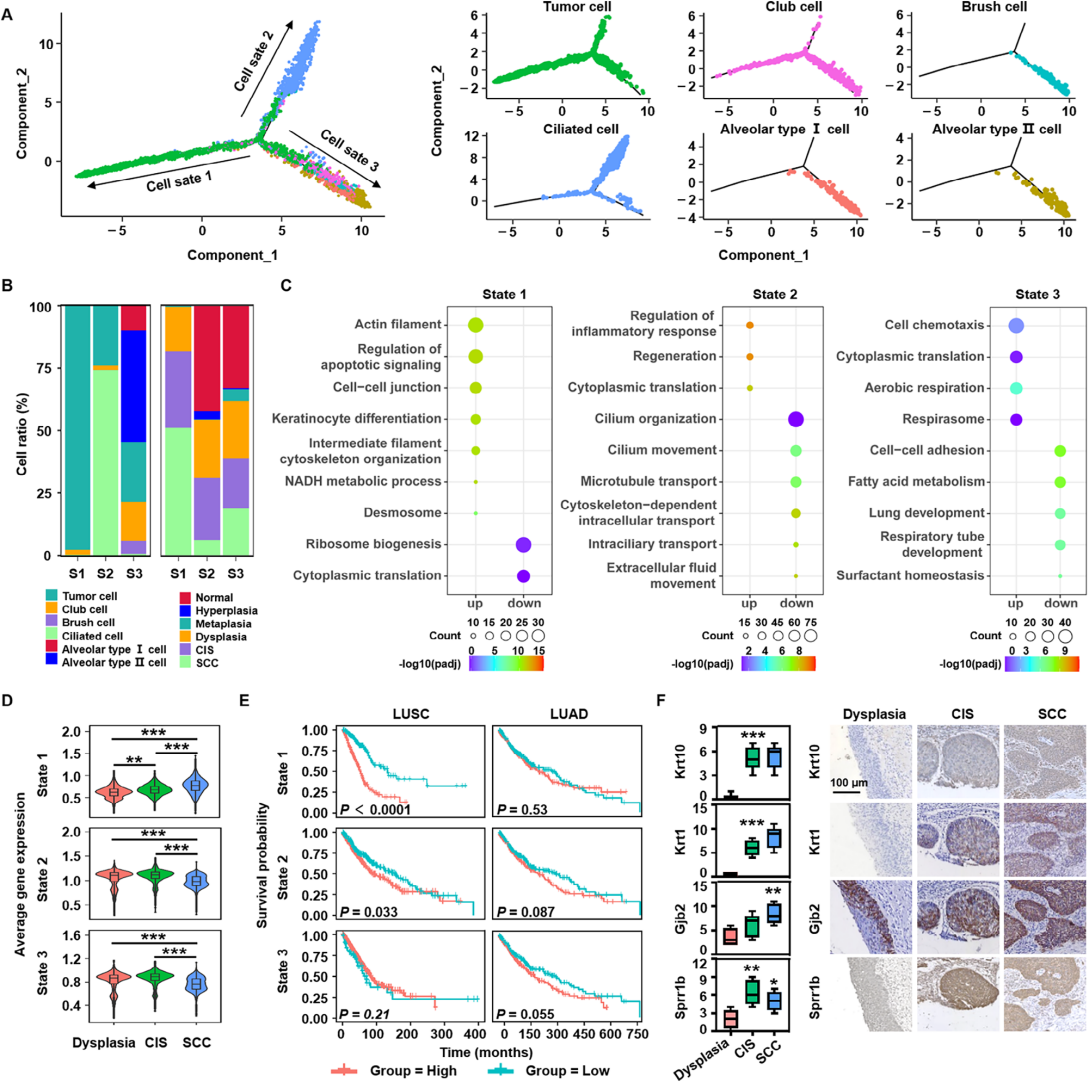
Identification of a novel tumor cell signature in LUSC. A, Unsupervised transcriptional trajectory of normal and malignant epithelial cells from Monocle 2, colored by cell state and subset. B, Relative ratios of cell subsets and pathological stages for each cell state shown in (A). C, Functional categories (GO terms) of signature genes specific to each cell state, as shown in (A). D, Changes in the average gene expression of each state‐specific signature for dysplastic, CIS, and SCC lesions. E, Kaplan–Meier OS curves from TCGA data in LUSC (*n* = 501 samples) and LUAD (*n* = 526 samples) patients. F, (Left) Box plots depicting the quantified protein levels of selected markers specific to State 1. Each group includes six biologically independent samples (*n* = 6). (Right) Representative IHC staining of Krt10, Krt1, Gjb2, and Sprr1b in dysplasia, CIS, and SCC samples. Scale bar, 100 µm. *p < 0.05, **p < 0.01, ***p < 0.001; two‐sided Wilcoxon test (D), two‐sided log‐rank test (E), and one‐way ANOVA (F) were used.

Based on the potential co‐expressed genes and their corresponding cis‐regulatory elements identified via SCENIC,^[^ ^23^ ^]^ the Monocle 2 algorithm was used to reconstruct the transcriptional trajectories of various epithelial and tumor cells.^[^ ^24^ ^]^ As shown in **Figure** **4**A, the two termini of the pseudospace tree populated by ciliated and alveolar cells revealed distinct differentiation paths of the proximal and distal epithelial cell lineages. Club cells were distributed throughout the trajectory of ciliated and alveolar cells (Figure 4A), yet exhibited higher CNV levels than the terminally differentiated populations (Figure S8E, Supporting Information). Stage‐resolved transcriptomic profiling further identified dynamic molecular regulators governing xenobiotic metabolism, immune response, proliferation, and apoptosis (Figure S13, Supporting Information), thereby offering insights into genomic instability potentially associated with oncogenic transformation. If the three different trajectory branches were defined as distinct cell states, we found that tumor cells may be present in all three cell states. However, when the relationships between all epithelial cell subtypes and the cell state were further analyzed, our data revealed that the cell State 2 and State 3 branches directed the cell fate toward the normal epithelium, and cell State 1 indicated the opposite ends of cell State 2 and State 3 (Figure 4A). The proportion of tumor cells in State 1, predominantly derived from CIS and invasive SCC lesions, was significantly greater than that in the other two states (Figure 4B).

To reveal the potential molecular mechanisms in the different states, we further searched for differentially expressed genes closely related to cell fate. Compared to normal epithelial cells, tumor cells presented 23, 34, and 59 upregulated genes and 27, 77, and 55 downregulated genes in States 1, 2, and 3, respectively (Table S3). Gene ontology (GO) analysis revealed that the differentially expressed genes involved in States 2 and 3 were primarily related to cilium motility, protein synthesis and transport, and respiratory system development (Figure 4C). Conversely, the differentially expressed genes involved in State 1 were involved in keratinization, intermediate filament organization, and gap junctions, which are classic features of SCC (Figure 4C). Compared to those in States 2 and 3, the cells in State 1 were more likely to deviate from normal differentiation. Furthermore, the expression levels of State 1‐specific genes increased in epithelial cells with lesion progression (Figure 4D). This finding was corroborated using external datasets (GSE33479 and GSE73402) related to human lung squamous carcinogenesis (Figure S14A, Supporting Information). We evaluated the clinical impact of the three‐state signatures using data from The Cancer Genome Atlas (TCGA) and independent cohorts. Patients with LUSC in both cohorts with high gene expression levels of State 1 signatures had significantly worse overall survival (OS) than those with low expression levels. In contrast, no significant difference in survival was observed in lung adenocarcinoma (LUAD) cases (Figure 4E, Figure S14B, Supporting Information), further confirming the distinct involvement of State 1 in LUSC development. Moreover, the top four genes in State 1 (Krt10, Krt1, Gjb2, and Sprr1b) were validated to be upregulated in dysplastic, CIS, and SCC lesions (Figure 4F).

### 2.4 Activation and Perturbation of T‐Cell Immunity During LUSC Development

**Figure 5:**
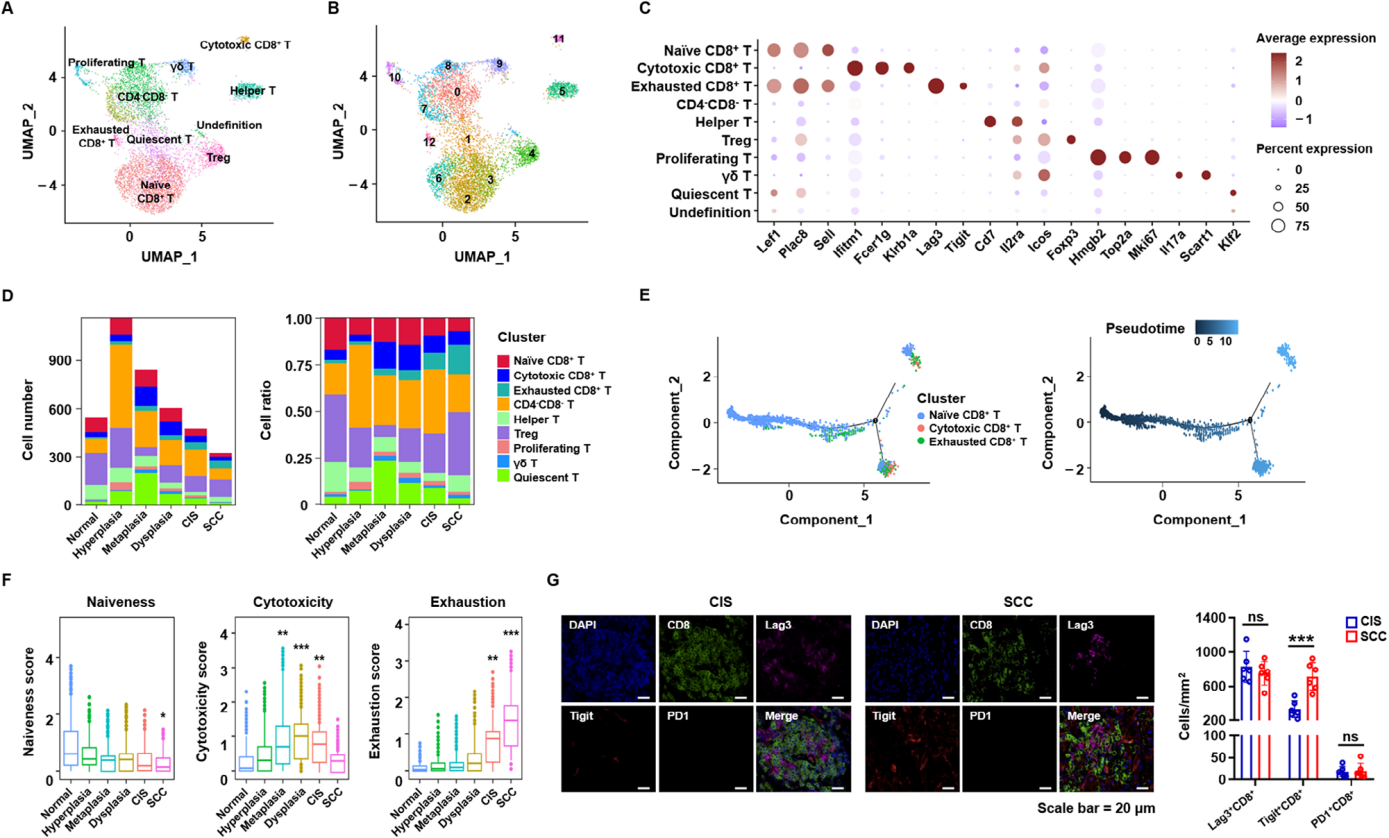
T‐cell‐mediated immune responses in the development of LUSC. A‐B, Diverse T cells from 10 samples, with major cell subtypes (A) and subtype‐corresponding subclusters (naïve CD8^+^ T cells, including 2, 3, and 6 subclusters; CD4^−^CD8^−^ T cells, including 0, 7, and 8 subclusters (B)). C, Average expression of specific markers in each cell type depicted in (A). D, Average cell number and relative ratio of T‐cell subsets from lung tissues at each stage (excluding undetermined cells). E, Unsupervised trajectory analysis of T‐cell functional states, colored by cell population and pseudotime. F, T‐cell functional features in the lung tissues of each stage. The scores of naiveness, cytotoxicity, and exhaustion were calculated according to the mean expression levels of signature genes. G, (Left) Representative multiple immunofluorescence images of Lag3, Tigit, and PD1 on CD8^+^ T cells in CIS and SCC lesions. Scale bar, 20 µm. (Right) Percentage of Lag3^+^, Tigit^+^, and PD1^+^ CD8^+^ T cells per mm^2^ in different groups. At least three separate fields from each rat were quantified. *p < 0.05, **p < 0.01, ***p < 0.001; ns, not significant; one‐way ANOVA with Tukey's post hoc test (F) and two‐tailed Student's t‐test (G) were used.

As key components of the adaptive immune system, T cells have diverse functions that contribute to both antitumor immunity and immunosuppression, thereby controlling tumorigenesis.^[^ ^25^ ^]^ To reveal the diversity of the T‐cell lineage and functional status, we performed subclustering analysis on 4656 T cells from all the samples and identified nine distinct cell types based on the well‐established expression patterns of characteristic marker genes: naïve CD8^+^ T, cytotoxic CD8^+^ T, exhausted CD8^+^ T, CD4^−^CD8^−^ T, helper T, regulatory T (Treg), proliferating T, gamma delta (γδ) T, and quiescent T cells (**Figure** **5**A–C). The proportion of Tregs gradually decreased from normal lesions to hyperplastic and metaplastic lesions, but increased from dysplastic lesions to CIS and SCC lesions. The number of cytotoxic CD8^+^ T cells transiently increased during premalignant stages, such as metaplasia, whereas exhausted CD8^+^ T cells emerged in large numbers once cancer lesions formed (Figure 5D).

To elucidate the immune transition of CD8^+^ T cells, we conducted an unsupervised trajectory analysis of naïve, cytotoxic, and exhausted CD8^+^ T cells using the Monocle 2 algorithm. The results of the trajectory analysis revealed that naïve T cells may differentiate into cytotoxic and exhausted CD8^+^ T cells (Figure 5E). In normal lungs, and at each stage of lesion development, scores for naiveness, exhaustion, and cytotoxicity were calculated based on the mean expression of the signature genes. During LUSC development, T‐cell function ranged from functional activation to functional exhaustion (Figure 5F). In addition to PD1, Lag3 and Tigit have also become increasingly important immune checkpoint molecules in T‐cell exhaustion.^[^ ^26^ ^]^ We also observed high Lag3 and Tigit expression in the CD8^+^ T cells of both CIS and SCC (Figure 5G).

### 2.5 Myeloid Cells Predict Immune Suppression Status in the Tumor Microenvironment

**Figure 6:**
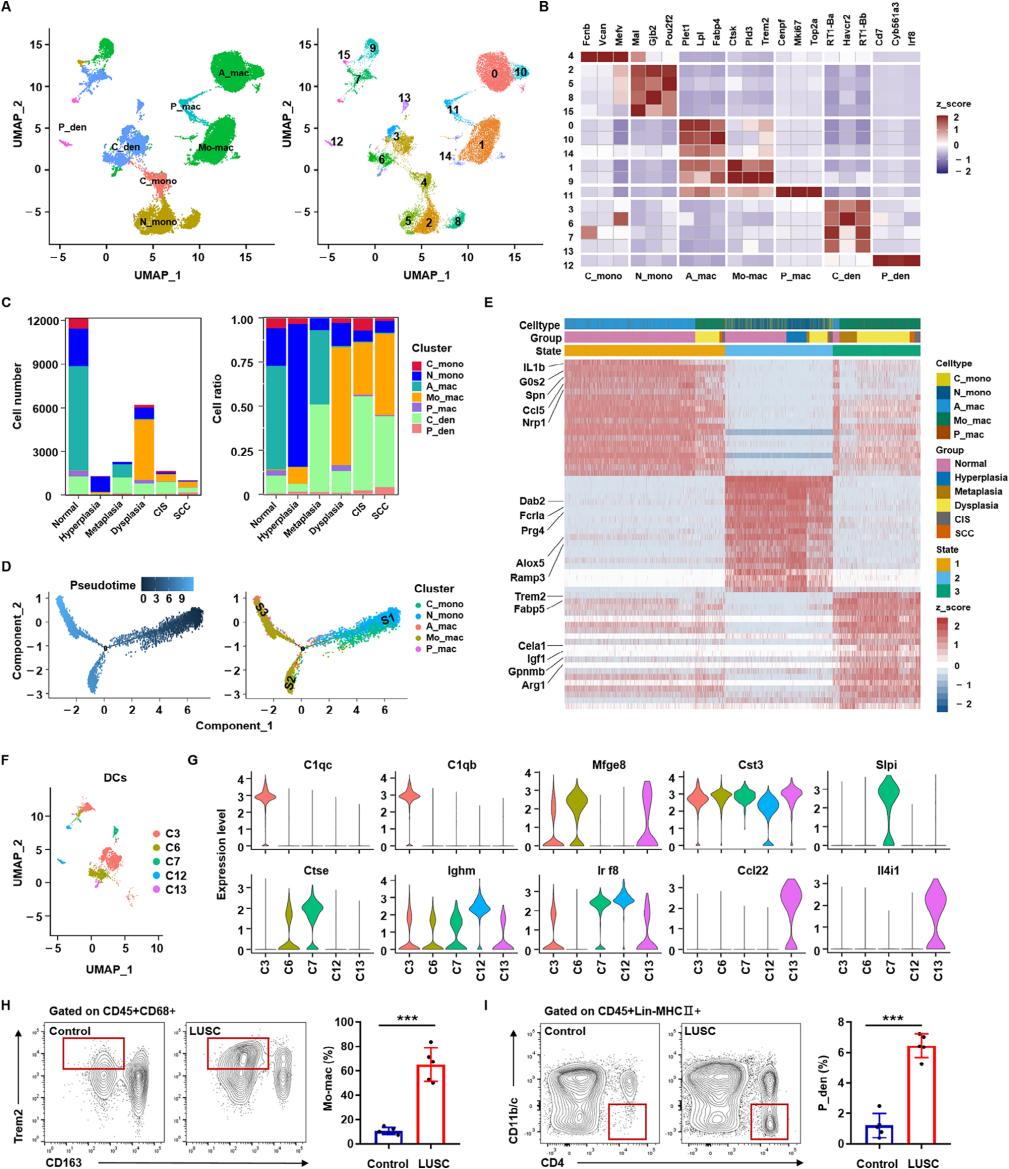
Relationships between myeloid cells and the immune microenvironment. A, Diverse myeloid cells from 10 samples, color‐coded by major cell subtypes (left) and subtype‐corresponding subclusters (N‐monos including subclusters 2, 5, 8, and 15; Mo‐macs including subclusters 1 and 9 (right)). B, The relative expression levels of canonical marker genes (top 3) in each subset. Subclusters are shown in columns, and genes are shown in rows. The mean expression levels were scaled by mean‐centering and transformed to a scale from −2 to 2. C, Average cell number and relative ratio of myeloid cell subtypes from each stage. D, Unsupervised trajectory of monocyte and macrophage state transitions. The branched trajectory is colored according to the cell state, cell subset, and pseudotime. E, Heatmap of the top 20 upregulated genes specific to the cell state shown in (D). The mean expression levels were scaled by mean‐centering and transformed to a scale from ‐2 to 2. F, Five color‐coded DC subpopulations from 10 samples. G, Expression levels of 10 selected marker genes across 5 DC subpopulations as indicated in (F). H, (Left) Representative flow cytometry plots showing Mo‐mac (CD45+CD68+CD163‐Trem2+) populations in normal lung and LUSC tissues. (Right) Comparison of the percentages of Mo‐macs between normal lung and LUSC tissues (*n* = 5). I, (Left) Representative flow cytometry plots showing P_den (CD45+Lin‐MHCII+CD11b/c‐CD4+) populations in normal lung and LUSC tissues. (Right) Comparison of the percentages of P_dens between normal lung and LUSC tissues (*n* = 5). ***p < 0.001; two‐tailed Student's *t*‐test was used (H, I). C_mono: classical monocyte; N_mono: nonclassical monocyte; A_mac: alveolar macrophage; Mo_mac: monocyte‐derived macrophage; P_mac: proliferating macrophage; C_den: conventional dendritic cell; P_den: plasmacytoid dendritic cell.

Myeloid cells, a diverse set of immune cells, play important regulatory roles in the tumor microenvironment.^[^ ^27^ ^]^ By subclustering 24501 myeloid cells, 16 distinct myeloid cell clusters were identified: five monocyte clusters, six macrophage clusters, and five dendritic cell (DC) clusters (**Figure** **6**A). Classical monocytes (C‐monos) were characterized by high expression levels of Fcnb, Vcan, Mefv, and Mal, whereas nonclassical monocytes (N‐monos) showed high expression levels of Mal, Gjb2, and Pou2f2 (Figure 6B). There was an influx of N‐monos at the hyperplasia stage, followed by a sharp decline in its abundance in the more severe lesion stages (Figure 6C). Further GO enrichment analysis revealed that N‐monos was enriched for biological processes related to inflammatory response, chemokine activity, and humoral immune response (Figure S15, Supporting Information).

According to the expression spectrum of characteristic genes (Plet1, Lpl, Fabp4, Ctsk, Pld3, Trem2, Cenpf, Mki67, and Top2a), macrophages were further subdivided into alveolar macrophages (A_macs, Clusters 0, 10, and 14), monocyte‐derived macrophages (Mo‐macs, Clusters 1 and 9), and proliferating macrophages (P_macs, Cluster 11) (Figure 6A,B). A_macs in normal lung tissues and metaplasia samples were high in weight, whereas the proportion of Mo_macs increased at the dysplasia stage and then remained relatively stable at a high level (Figure 6C). Trajectory analysis based on the SCORPIUS algorithm revealed that Mo‐macs presented clearly different molecular spectra (such as Ctsk, PId3, and Trem2) from A_macs, leading to the adaptation of malignant cells to the immune response (Figure S16, Supporting Information). To infer the macrophage differentiation pathway, transcriptional differential analysis from monocytes to macrophages was performed using an unsupervised trajectory (Figure 6D, Table S4, Supporting Information). Genes associated with inflammatory response, cell migration, and T‐cell co‐stimulation, including IL1b, Dab2, Alox5, Nrp1, and Spn, were significantly downregulated. Conversely, genes involved in cholesterol metabolism, phagocytic vesicle formation, and negative regulation of TNF production, such as Trem2, Fabp5, Cela1, Igf1, Gpnmb, and Arg1 were significantly upregulated along the pseudotime axis (Figure 6E). These data indicate that tumor‐associated macrophages in LUSC predominantly originate from Mo‐macs with unique gene expression patterns.

DCs are a rare immune cell population that play a critical role in initiating and regulating adaptive immune responses in tumors.^[^ ^28^ ^]^ Conventional DCs (C_dens) showed high expression of RT1‐Ba, Havcr2, and RT1‐Bb, whereas plasmacytoid DCs (P_dens) showed elevated expression of Cd7, Cyb561a3, and Irf8 (Figure 6B). In our study, C_dens was further divided into Clusters 3, 6, 7, and 13, whereas Cluster 12 represented the P_den subtype, which expressed various marker genes, including C1qc, C1qb, Mfge8, Cst3, Slpi, Ctse, Ighm, Irf8, Ccl22, and Il4i1 (Figure 6F,G). To investigate the potential intercellular communication, we performed Connectome analysis to acquire cell–cell signaling links (Figure S17A,B, Supporting Information). In the VEGF signaling pathway network, plasmacytoid DCs and Mo‐macs took over nonclassical monocytes as the lesions advanced (Figure S17C, Supporting Information). Additionally, Mo‐macs and plasmacytoid DCs played a predominant role in the TGFβ and CXCL pathway networks, which are implicated in epithelial‐mesenchymal transition, angiogenesis, and immunosuppression (Figure S17D, Supporting Information). Furthermore, flow cytometry revealed that the proportions of Trem2^+^ Mo‐macs and plasmacytoid DCs were elevated in SCC tissues (Figure 6H,I). Collectively, these data suggest that Mo‐macs and plasmacytoid DCs are involved in LUSC carcinogenesis and progression.

## 3 Discussion

Over the past several decades, despite great advances in targeted therapy and immunotherapy, as well as early detection of lung cancer, the prognosis for patients with LUSC has remained dismal. The 5‐year OS of patients with distant metastases is approximately 15%, whereas for those with local disease, the 5‐year OS can reach approximately 51%.^[^ ^29^ ^]^ Furthermore, prior to the development of malignant lesions, a series of signaling and transcriptional/molecular aberrations, such as increased oxidative phosphorylation,^[^ ^30^ ^]^ strong chromosomal instability^[^ ^14^ ^]^ and loss of epithelial polarity,^[^ ^31^ ^]^ have been identified. Thus, a deep and comprehensive understanding of cancer precursor biology might not only improve early diagnosis and reduce overtreatment but also foster preventive interventions targeting early pathogenic clones in LUSC.

Smoking is widely acknowledged as the best‐known risk factor for LUSC.^[^ ^32^, ^33^ ^]^ To replicate this process in vivo, two tobacco‐derived chemical compounds, DEN and 3‐MCA, were used to induce autochthonous tumors in the bronchi. Unlike other models that employ only a single carcinogen^[^ ^34^, ^35^, ^36^, ^37^, ^38^, ^39^ ^]^ or adopt intraperitoneal injection^[^ ^40^, ^41^ ^]^ with time spans ranging from 9 weeks to 17 months, the combined use of 3‐MCA and DEN can shorten the induction process of lesions to approximately 1 month, and demonstrate the stepwise histological progression of LUSC, saving clinical research time and costs. In humans, due to extensive immune heterogeneity and diverse lifestyles, some precancerous lesions may progress further, whereas others may spontaneously regress.^[^ ^42^ ^]^ In an induced animal model, transformation of precancerous lesions into cancerous lesions is inevitable as long as the carcinogen persists.^[^ ^43^ ^]^ By monitoring tumor development over time, we observed the continual proliferation of epithelial cells, which was characterized by the transition from a normal pseudostratified morphology to various lesions.^[^ ^44^ ^]^ Consistent with previous cross‐sectional studies indicating the activation of immune evasion from high‐grade preinvasive lesions,^[^ ^11^, ^12^, ^45^ ^]^ we confirmed a transitory increase in immune cells, including T, B, and NK cells, at the hyperplasia stage, followed by gradual depletion from metaplasia to SCC. These findings suggest that to reduce the occurrence of LUSC, early immune intervention should be provided to high‐risk populations, and it is important to eliminate the carcinogenic environment as early as possible, such as by smoking cessation.

LUSC is characterized by marked aneuploidy with somatic copy number alterations across the genome.^[^ ^46^ ^]^ Genome‐wide copy number alterations are better indicators of tumor relatedness than traditional histology.^[^ ^47^ ^]^ A large proportion of canonical CNVs was detected in the hyperplasia/metaplasia stages, and their frequency increased with lesion progression. These chromosomal aberrations correspond to the location of the tumor suppressor gene TP53, the driver gene FGFR2, and its downstream effector gene CCND1, which are well‐known regulators of the cell cycle and apoptosis,^[^ ^48^ ^]^ suggesting their crucial roles in tumor initiation from the beginning. Similarly, frequent early occurrences and persistent chromosomal instability have been reported in other models,^[^ ^49^ ^]^ albeit with variations in location and genes. Additionally, our analysis revealed a range of noncanonical CNV patterns in SCC, including 3p gain and 7q loss. Genomic aberrations at these loci, including Itga6, Hspa5, and Btg1, are associated with abnormal proliferation, invasion, and poor prognosis.^[^ ^50^, ^51^, ^52^ ^]^ Epithelial subclones with high CNV scores first emerged at the pathomorphologically recognized dysplasia stage. Given that the detection of dysplastic lesions cannot be performed by routine imaging examinations, such as CT or PET,^[^ ^53^ ^]^ future CNV evaluation from plasma cell‐free DNA could assist in identifying high‐risk patients with premalignant lesions.

Single‐cell RNA‐seq has led to a rapid growth in prediction methods to infer the precise sequence of gene regulatory events that promote the transition from one cell state to another.^[^ ^24^ ^]^ Although epithelial cells in the bronchi were heterogeneous, we demonstrated that they exhibited three cell states during the evolution of malignant cells from normal to SCC. Among these cell states, tumor cells in States 2 and 3 were dysregulated during respiratory system development and metabolic pathways, whereas tumor cells in State 1 were closely related to squamous differentiation and anti‐apoptotic signaling. Studies have revealed activation of metabolic pathways in the airways of participants with dysplastic lesions^[^ ^30^ ^]^ and inactivation of cilia‐associated pathways in hyperplastic lesions.^[^ ^11^ ^]^ Furthermore, squamous differentiation and the PI3K/Akt pathway are unlikely to play roles in the earliest developmental stages.^[^ ^54^ ^]^ Therefore, the regulatory genes involved in States 2 and 3 represent early events, whereas those involved in State 1 represent late events in the malignant transformation of LUSC. The positive correlation between high expression of State 1‐specific genes and poor prognosis further supports this perspective. Sprr1b, Gjb2, Krt1, and Krt10, which are differentially expressed in State 1, are associated with structural proteins encoded by the cornified cell envelope,^[^ ^55^ ^]^ gap junction,^[^ ^56^ ^]^ and intermediate filament;^[^ ^57^ ^]^ however, their detailed roles in epithelial proliferation, differentiation, and apoptosis contributing to oncogenesis are yet to be elucidated and require further investigation. Additionally, as bronchiolar tissue‐specific stem cells and potential tumor‐initiating cells,^[^ ^58^ ^]^ club cells are distributed at the root of the pseudospace trajectory and exhibit three branches shared with the tumor cells. Hence, it is presumed that the club cell subset in State 1 is susceptible to oncogenic transformation, presenting an intriguing avenue as a potential therapeutic target.

Understanding the largely unknown biology of carcinogenesis requires identifying the determinants of intralesional immune reactions during cancer development. We confirmed that immune sensing occurred at the earliest stage of transformation, which was supported by the fact that from the initiation of carcinogen stimulation, the number and composition of T cells were oriented toward active responses. As exposure to carcinogens continues, immunosuppression gradually develops. High‐grade lesions contain a greater number of Tregs, which may hinder the recruitment and effector function of CD8^+^ T cells.^[^ ^59^ ^]^ Recently, anti‐PD1 therapeutics have received increasing attention for reinvigorating the activity of CD8^+^ T cells.^[^ ^60^ ^]^ We observed a co‐increase in Lag3 and Tigit, rather than PD1, in exhausted CD8^+^ T cells, which may provide new insights into the limited clinical efficacy of PD1 blockade.^[^ ^61^ ^]^

Myeloid cells, which are crucial components of the tumor microenvironment,^[^ ^62^, ^63^ ^]^ are critically involved in the regulation of carcinogenesis. We identified three distinct macrophage populations. Most tumor‐associated macrophages were Mo‐macs, which expressed high levels of Trem2. Previous single‐cell RNA sequencing studies show that both human and murine lung tumors are deprived of NK cells and heavily infiltrated by Mo‐macs.^[^ ^64^, ^65^, ^66^ ^]^ Trem2^+^ Mo‐macs suppress the recruitment and activation of NK cells by disabling IL‐18 signaling.^[^ ^67^ ^]^ Furthermore, genetic ablation of Trem2 or treatment with an anti‐Trem2 mAb can inhibit tumor growth and promote regression when combined with immune checkpoint blockade.^[^ ^68^, ^69^, ^70^ ^]^ We also observed that Trem2^+^ Mo‐macs were more abundant in late developmental stages than in early stages, providing a new Mo‐mac‐mediated immunotherapy paradigm. Notably, among the different subclusters, DCs presented high heterogeneity. Although the cell populations did not align with classical typing, in which conventional DCs are classified into two major subtypes, cDC1s and cDC2s,^[^ ^62^ ^]^ we identified plasmacytoid DCs as a specific subpopulation. However, the function of plasmacytoid DCs remains controversial, with studies indicating their contribution to immunosuppression,^[^ ^71^, ^72^, ^73^ ^]^ whereas the production of IFNα/β and TNFα indicates their antitumor potential.^[^ ^74^ ^]^ Further validation through well‐designed trials and clinical studies is necessary to elucidate the function of plasmacytoid DCs in tumor carcinogenesis.

Overall, our study delineates a complex biological evolutionary trajectory of the bronchial epithelium and the corresponding dynamic gene alterations in immune cells within a series of lesions during LUSC development. These findings not only deepen our understanding of the occurrence and development of LUSC, but also provide some possible therapeutic paradigms, such as those that target exhausted CD8^+^ T cells, Mo‐macs, or plasmacytoid DCs.

## 4 Experimental Section

### Establishment of the LUSC Model

Female Wistar rats (8 weeks old, 200 ± 20 g) were housed in a pathogen‐free environment at the Animal Center of the First Affiliated Hospital, Sun Yat‐sen University. The rats were fasted overnight prior to instillation and deprived of water on the day of the experiment. To increase the tumorigenic effect, iodized oil was used to dissolve the chemical carcinogens 3‐MCA (100 mg mL^−1^, Sigma) and DEN (95 mg mL^−1^, Sigma) at 65–70 °C overnight. Basal anesthesia was induced through the intraperitoneal injection of 150 µL of 3% sodium pentobarbital solution (Sigma), followed by inhalation administration of isoflurane (RWD) to achieve deep anesthesia. Subsequently, the rats were randomly assigned to six groups (*n* = 8). In each group, six rats were instilled with 100 µL of carcinogen‐containing iodized oil (Dknano) into the left lower lobe through a special blunt needle, while the remaining two rats were administered 100 µL of iodized oil without carcinogens as a control. The success of the instillation was confirmed by micro‐CT (Bruker). To prevent infection, drinking water containing 1 g L^−1^ ampicillin (Sangon Biotech) and 1.5 g L^−1^ streptomycin (Sangon Biotech) was provided from days 2 to 15 post‐instillation. The rats were euthanized at various time points (days 0, 7, 14, 21, 28, and 35) following instillation. Lung tissues from the left lower lobe were excised and a portion of the tissue was processed for H&E staining. The remaining tissue was used for single‐cell suspension preparation. All animal experiments were approved by the Institutional Animal Care and Use Committee of Sun Yat‐sen University.

### Immunohistochemistry (IHC)

The slides, with a thickness of 4 µm, were deparaffinized using xylene and graded ethanol. EDTA or citrate buffer was used for the antigen retrieval. The slides were subsequently incubated in 3% hydrogen peroxide (H_2_O_2_) to quench endogenous peroxidase activity and then incubated overnight at 4 °C with the following primary antibodies: anti‐Krt10 (polyclonal, ZSGB‐BIO), anti‐Krt1 (EPR17744, Abcam), anti‐Gjb2 (ab65969, Abcam), anti‐Sprr1b (polyclonal, Proteintech), anti‐CK5 (EP1601Y, Abcam), anti‐P63 (EPR5701, Abcam), anti‐TTF1 (EP1584Y, Abcam), and anti‐CK7 (EPR17078, Abcam). Finally, a chromogenic reaction was developed using diaminobenzidine (DAB), followed by counterstaining with hematoxylin. IHC images were obtained using the KFPRO‐005 digital platform (Jiang Feng). The IHC score was calculated by multiplying the number of positive cells with the staining intensity. The number of positive cells was graded using a five‐category system as follows: 0, 0% to 5% stained cells; 1, 6% to 25% stained cells; 2, 26% to 50% stained cells; 3, 51% to 75% stained cells; and 4, > 75% stained cells. The staining intensity was graded using a four‐category system as follows: 0, no staining; 1, weak staining; 2, moderate staining; and 3, strong staining. All histological evaluations were independently conducted by two professional pathologists and discrepancies were resolved by consensus discussion.

### Sample Preparation

Fresh rat lung tissues were dissected and dissociated using a tissue dissociation kit (Miltenyi Biotech, Germany). The tissue fragments were transferred to a C tube containing an enzyme mixture (including deoxyribonuclease I, dispase, collagenase D, and collagenase II) and incubated for 30 min at 37 °C on the MACSmix tube rotator. The C tube was subsequently inverted on a GentleMACS separator and the GentleMACS program m_lung_01 was run twice. The cell suspension was filtered through a 70‐µm filter. Dead cells and cell debris were removed using Ficoll‐Paque PLUS (GE Healthcare). Finally, the cells were stained with acridine orange (AO)/propyl iodide, and the number and activity were detected using a fluorescent cell counter (Arthur, NanoEntek, Korea).

### Single‐Cell Capture, Library Preparation, and Single‐Cell RNA Sequencing

Single cells were captured using a Chromium Controller (10× Genomics) and processed to generate gel bead‐in‐emulsion (GEM) products. The libraries were established via the Chromium Single‐cell 3′ Library and Gel Bead Kit V2 (10x Genomics), with a target of 5000 cells and 350 million reads. Each sample was processed on an independent chromium single‐cell A‐chip (10x Genomics). Libraries were pooled and sequenced on an Illumina HiSeq2500 platform.

### Data Processing and Dimension Reduction

Raw sequencing data were processed using CellRanger and imported into R using the Seurat package (V4.3.0) for analysis. For the same data subsets, the enrollment criteria must meet the following request: the number of genes that could be detected in each cell must be ≥ 200, and the number of cells in which each gene could be detected must be ≥ 3. The R package DoubletFinder (v2.0.3) was used to exclude potential doublets and occasional higher‐order multiplets from the datasets. The harmony method was applied to integrate cells from different sequencing datasets. The top 2000 highly variable genes were generated and used for principal component analysis (PCA). Based on the PCA elbow plot, clustering was performed on the integrated assay using the FindClusters() function with 30 PCs at a resolution of 0.6. The clusters were further visualized and explored using UMAP and tSNE plots.

### Identification of Differentially Expressed Genes

The log2‐fold change (log2FC) between different groups was manually calculated using Seurat FindMarkers. The significance of the differences was determined by applying a two‐sided Student's *t*‐test with Bonferroni correction. The signature genes were then screened based on statistical benchmarks, including an absolute log2FC > 0.25 and an adjusted *p* value (Bonferroni) < 0.05. In addition, signature genes must be expressed in more than 25% of the cells belonging to the target cluster. The top differentially expressed genes in each cluster were selected for in situ analysis to confirm cell type. Functional enrichment and pathway analyses of these differentially expressed genes were performed using ClusterProfiler and GSEA.

### CNV Inference and Clonality Analysis

Raw gene expression data were extracted from the Seurat object to construct a new gene‒cell matrix. The somatic large‐scale chromosomal CNV score of single cells was calculated using the R package, inferCNV (v1.17.0). To infer true tumor cells, T cells, macrophages, and endothelial cells from each sample were selected as reference cells. The inferCNV analysis was performed with default parameters (cutoff = 0.1; analysis_mode = “subclusters”; tumor_subcluster_partition_method = “random_trees”, denoise = TRUE, HMM = TRUE). Referencing the cytoband information from the RGSC 6.0, each p‐ or q‐arm‐level change was converted to an equivalent CNV based on its location. These CNVs were classified as either gains or losses using the inferCNV HMM‐based CNV prediction methods (i6 HMM). Subclones containing identical arm‐level CNVs were then collapsed, and phylogenetic trees were restructured to reflect the subclonal CNV architecture. For the automated generation of evolutionary trees, the UPhyloplot2 algorithm was used as previously described.^[^ ^21^ ^]^ Briefly, arm‐level CNV calls and the percentage of cells in each subclone were used as inputs. A scalable vector graphics (.svg) file visualizing the phylogenetic tree was generated for each sample. The arm length was proportional to the percentage of cells plus a spacer (circle diameter + 5 pixels).

### Inference of the Tumor Cell State and Immune Cell Development via Trajectory Analysis

Cell clusters from single‐cell RNA sequencing data were extracted and used to generate a trajectory using the Monocle algorithm (version 2.26.0). Briefly, the gene‒cell matrix was provided as an input to Monocle, and its newCellDataSet function was called to generate an object with the parameter expression Family = negbinomial.size. Highly variable genes were screened according to the following thresholds: mean_expression ≥ 0.1 and dispersion_empirical ≥ 1 * dispersion_fit. The structure of the trajectory was subsequently plotted in 2D space via the DDRTree dimensionality reduction algorithm, and the cells were ordered in pseudotime. The trajectory was inferred using the default parameters of Monocle. For the trajectory analysis of macrophage differentiation, the gene‒cell matrix at the scale of UMI counts was provided as an input to SCORPIUS (version 1.0.9). Eigen analysis was performed on the given matrix via the Spearman method using the following steps: 1) conduct k‐means clustering, 2) calculate a distance matrix between cluster centers via a custom distance function, 3) identify the shortest path connecting all cluster centers via the custom distance matrix, and 4) iteratively fit a curve to the given data via principal curves.

### Cell–Cell Interaction Analysis

Connectome (v 1.0.1) was used to construct the cell–cell communication network. Every cell parcellation was treated as a node, and the mean values of ligand and receptor expression were calculated by averaging across each cell parcellation and used to establish connections between nodes. Ligand‐receptor pairs were curated from the FANTOM5 database. Edge weights were computed using two complementary metrics: 1) mean‐wise connectivity, defined as the product of mean ligand expression in the sender cluster and mean receptor expression in the receiver cluster; and 2) proportional connectivity, quantified as the fraction of cells co‐expressing ligands and receptors across interacting clusters. To ensure biological relevance, edges were filtered by retaining only those ligand‐receptor pairs with z‐scores greater than 0.25 in both the sender and receiver clusters, and with expression levels detected in at least 10% of the cells within their respective clusters. Significant interactions were visualized as directed‐weighted graphs using CellChat (v. 2.1.2).

### Whole‐Genome Sequencing (WGS)

Tissue DNA was extracted using the DNeasy Blood & Tissue Kit (Qiagen, Hilden, Germany). The DNA purity was assessed using a NanoPhotometer P‐Class spectrophotometer (IMPLEN). Quantification was carried out with the Qubit dsDNA HS Assay Kit on the Qubit 3.0 Fluorometer (Thermo Fisher Scientific). Libraries were established via the NEB Next Ultra DNA Library Prep Kit for Illumina (NEB) following the manufacturer's instructions. The products were purified and enriched using polymerase chain reaction. The final libraries were evaluated using the KAPA Library Quantification Kit (KAPA Biosystems) and an Agilent 2100 Bioanalyzer. Paired‐end sequencing (2 × 150 bp) was performed using the Illumina NovaSeq 6000 platform.

### Survival Analysis

The RNA‐seq data of patients with LUSC and LUAD, comprising data from 501 LUSC tumors and 526 LUAD tumors, respectively, were downloaded from the UCSC Xena database. The log2 (norm_count + 1) scale was used to represent the expression level of each gene. To assess the prognostic value of the gene sets derived from the three cell states, tumor samples were categorized into two groups based on the mean expression levels of these genes. Survival curves were then generated via the Kaplan‒Meier formula implemented in the R package “survival,” and these curves were visualized via the ggsurvplot function provided by the R package “survminer.”

### Multiplex Immunofluorescence Staining

Multiplex immunofluorescence staining was performed using an Opal 7‐Color Manual IHC Kit (NEL811001KT). Briefly, the paraffin slides were deparaffinized using xylene and graded ethanol, followed by epitope retrieval, protein blocking, four‐cycle antibody incubation and staining, and DAPI counterstaining, in accordance with the manufacturer's instructions. The following primary antibodies were used: anti‐CD8 (Abcam, EPR6855, 1:400), anti‐PD1 (C81H6, CST), anti‐Lag3 (polyclonal, Abcam), and anti‐Tigit (EPR26037‐152, Abcam). Perkin Elmer Vectra (PerkinElmer, CLS140089) was used to scan the slides and obtain optical images for immunofluorescence analysis.

### Flow Cytometry

Frozen lung tissue specimens were thawed and enzymatically digested to obtain single‐cell suspensions as previously described. Cell suspensions were incubated for 30 min with fluorochrome‐conjugated antibodies targeting specific markers. The following antibodies were used for Mo‐macs phenotyping: anti‐rat PerCP‐eFluor 710‐CD45 (OX1, eBioscience), FITC‐CD68 (ED1, eBioscience), Alexa Fluor 647‐CD163 (ED2, Bio‐Rad), and PE‐Trem2 (Polyclonal, eBioscience). For plasmacytoid DCs phenotyping, the following antibodies were used: anti‐rat PerCP‐eFluor 710‐CD45 (OX1, eBioscience), FITC‐CD45RA (OX‐33, eBioscience), FITC‐CD3 (eBioG4.18 (G4.18), eBioscience), PE‐CD4 (OX35, eBioscience), BV421‐MHCII (OX‐6, BD), and APC‐CD11b/c (OX‐42, eBioscience). After staining, the cells were washed twice with PBS containing 1% BSA and resuspended in flow cytometry buffer for analysis.

## Conflict of Interest

The authors declare no conflict of interest.

## Author contributions

B.L., Y.L., S.C., T.Y., B.L. contributed equally to this work. B.H.L., Y.T.L., S.C.C., and T.T.Y. designed and performed all the experiments and analyzed the data. B.X.L., X.T.L., X.K.Z., T.T., and J.X.L. analyzed and interpreted the data. Q.R.Z., X.H.S., Z.P.X., and Y.X.W. provided pathological sections and conducted pathological evaluation. H.L.C., Z.Y., and Z.F.K. conceptualized and initiated the project and wrote the manuscript. Z.S.C. supervised and edited the article. All authors approved the submitted version.

## Supporting information



Supporting Information

Supplemental Table 1

Supplemental Table 2

Supplemental Table 3

Supplemental Table 4

## Data Availability

The RNA‐seq data has been deposited in Gene Expression Omnibus (GEO) under the accession number: GSE287159. Additional data is available upon resonable request.
