# Supplementary material for: Real‐Time Evolutionary Landscape of the Bronchial Epithelium and Corresponding Dynamic Immune Cell Alterations in Lung Squamous Cell Carcinogenesis
Source: Adv Sci (Weinh). 2025 Jun 5;12(31):e13256. doi: 10.1002/advs.202413256 (PMC12376527; doi:10.1002/advs.202413256)
Supplement: Supplementary file 1 — Supporting Information [file ADVS-12-e13256-s005.docx]

**Supporting Information**

**Real-time Evolutionary Landscape of the Bronchial Epithelium and Corresponding Dynamic Immune Cell Alterations in Lung Squamous Cell Carcinogenesis**

| **Contents** | **Page** |
| --- | --- |
| *Supplementary Figure 1* | 2 |
| *Supplementary Figure 2* | 3 |
| *Supplementary Figure 3* | 4 |
| *Supplementary Figure 4* | 5 |
| *Supplementary Figure 5* | 6 |
| *Supplementary Figure 6* | 7 |
| *Supplementary Figure 7* | 8 |
| *Supplementary Figure 8* | 9 |
| *Supplementary Figure 9* | 10 |
| *Supplementary Figure 10* | 11 |
| *Supplementary Figure 11* | 12 |
| *Supplementary Figure 12* | 12 |
| *Supplementary Figure 13* | 13 |
| *Supplementary Figure 14* | 13 |
| *Supplementary Figure 15* | 14 |
| *Supplementary Figure 16* | 15 |
| *Supplementary Figure 17* | 16 |


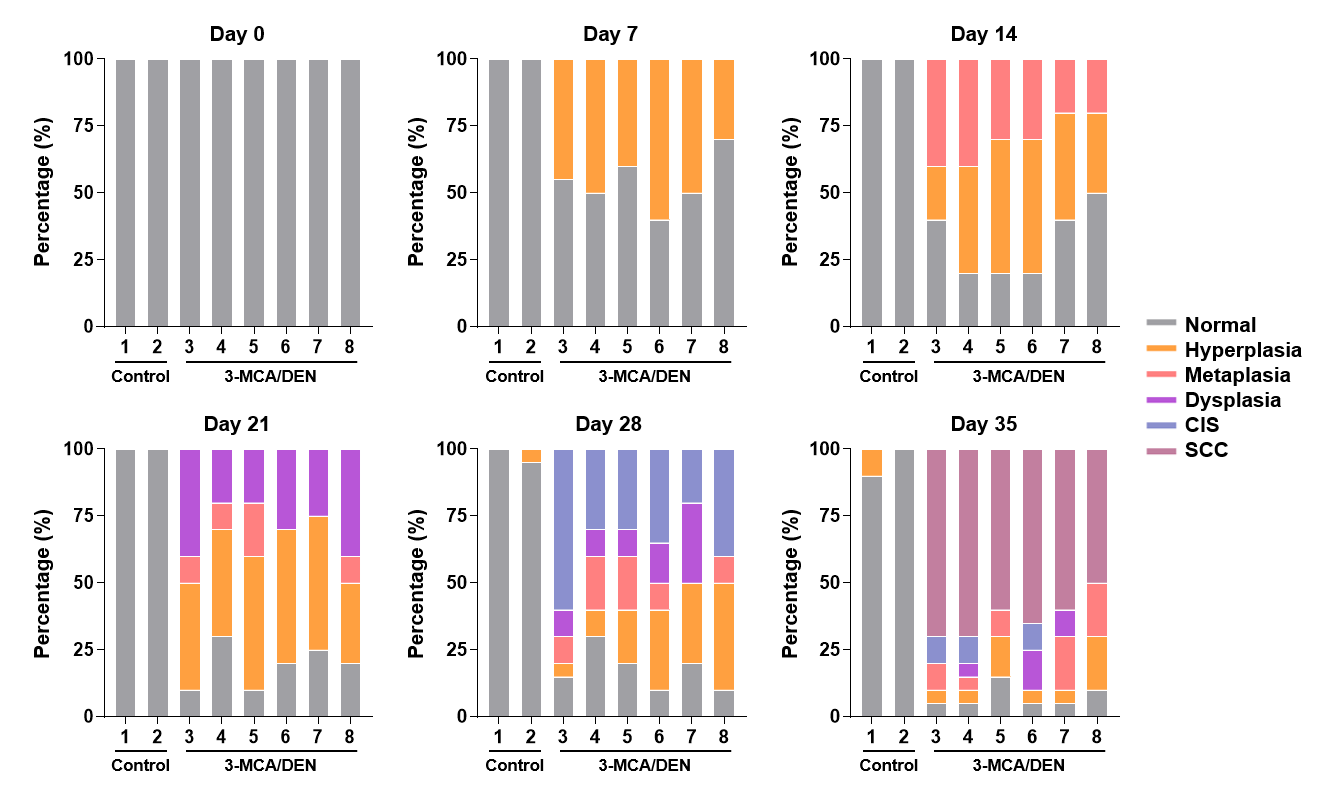


**Supplementary Figure 1 | Percentage of pathological phase of bronchial epithelium at each time point.** Rats were euthanized at days 0, 7, 14, 21, 28, and 35 following 3-MCA/DEN instillation. Lung tissues from the left lower lobe were resected and processed for H&E staining. At least two 4-μm-thick H&E sections (30 mm²/section) were quantified.


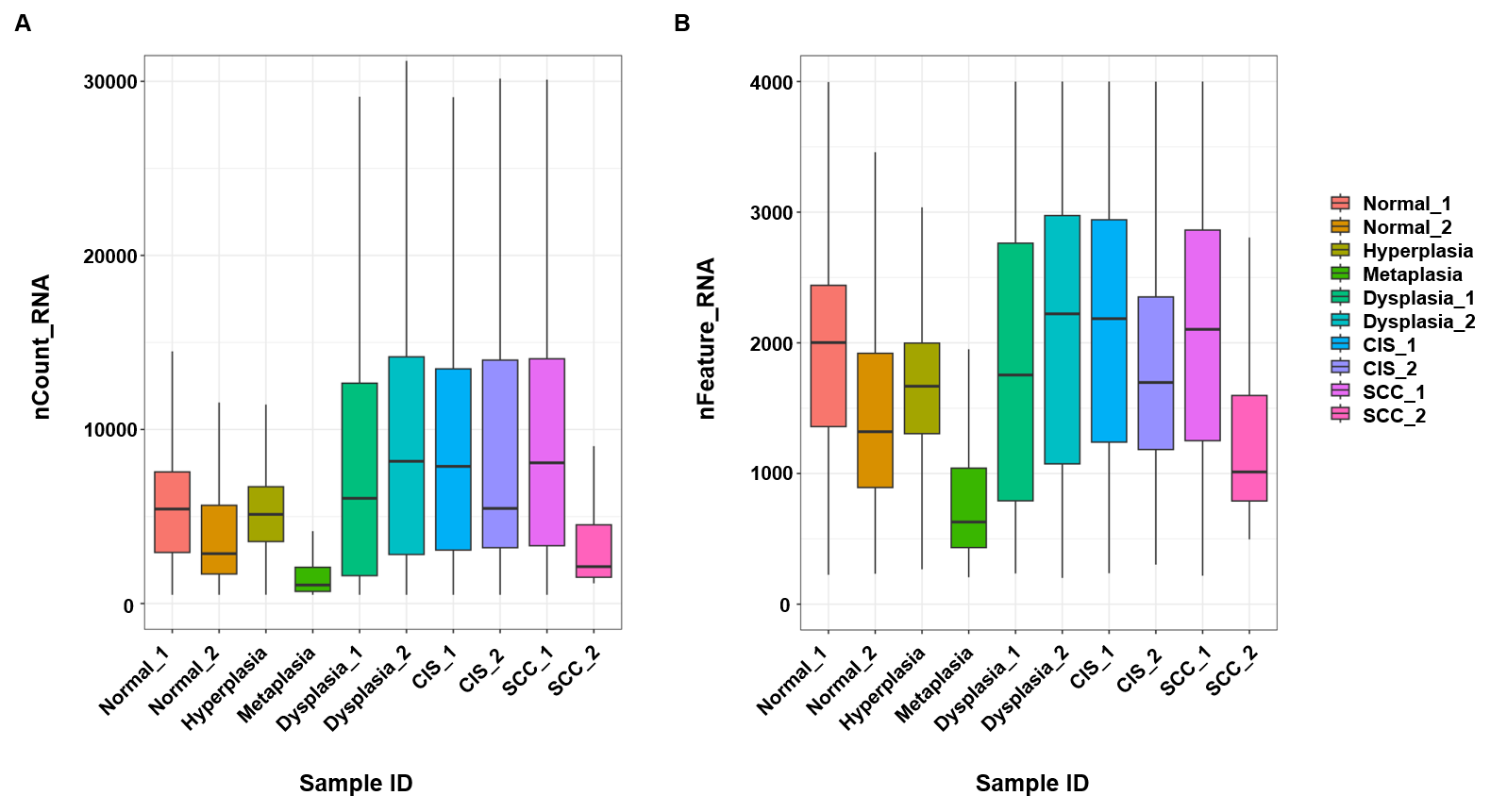


**Supplementary Figure 2 | Unique molecular indices and detected gene numbers in each sample.** **A-B,** The distribution of unique molecular indices (nCount_RNA) and detected gene numbers (nFeature_RNA) in single cells across all 10 samples: normal tissue (Normal_1 and Normal_2), hyperplasia, metaplasia, dysplasia (Dysplasia_1 and Dysplasia_2), carcinoma in situ (CIS_1 and CIS_2), and squamous cell carcinoma (SCC_1 and SCC_2) tissues. Each box represents the interquartile range (IQR, 25th to 75th percentile) with the median indicated by the horizontal line, and the whiskers cover 1.5 times the IQR. The sample stages are color-coded as indicated in the legend on the right.


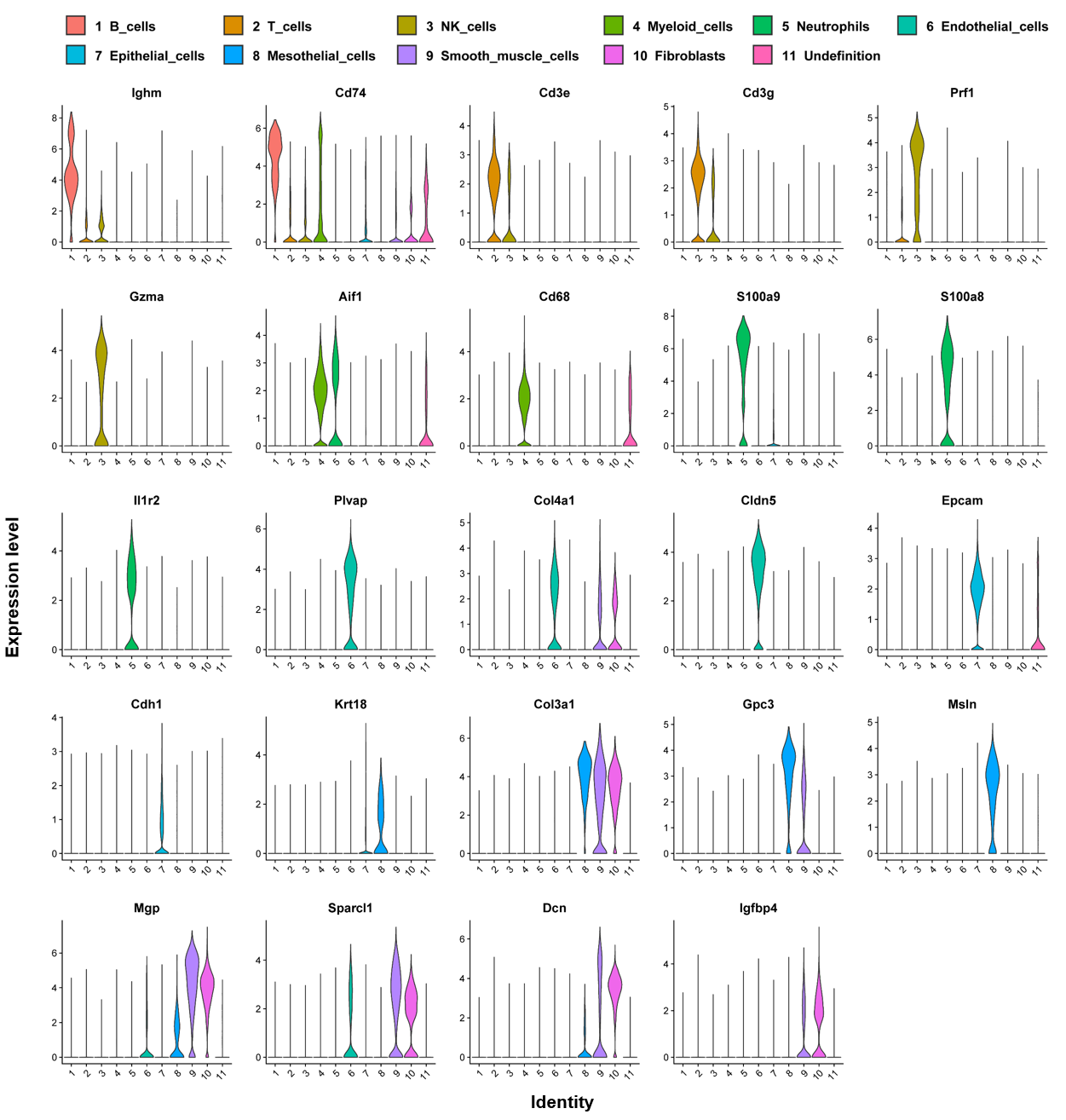


**Supplementary Figure 3 | Distribution and density of canonical markers across identified cell clusters.** The normalized expression levels of 24 specific signature genes for each of the 11 cell clusters. Each plot represents a single gene, with the y-axis indicating the expression level and the x-axis indicating the 11 cell clusters. The cell types are color coded in the top legend. The width of the violin plot indicates the density of cells with the same expression levels.


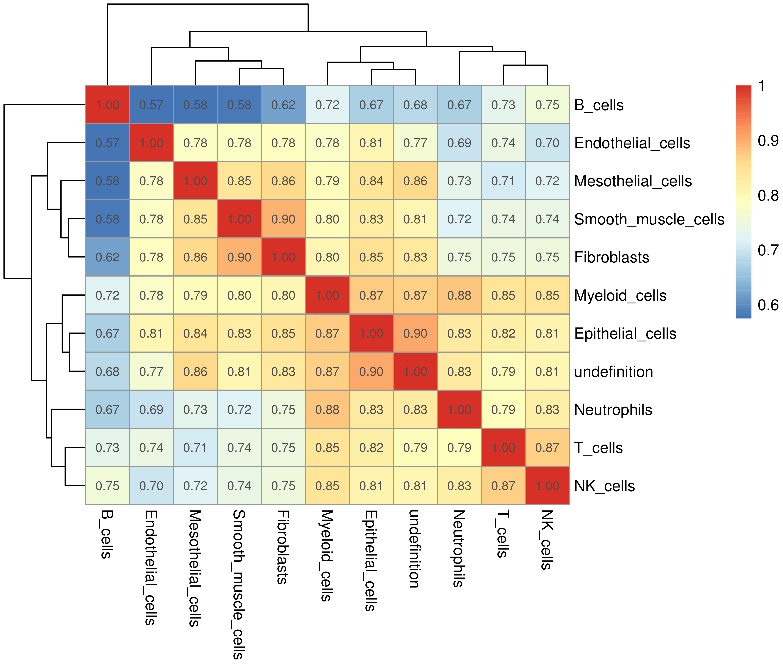


**Supplementary Figure 4 | Correlation analysis of average gene expression.** The correlation coefficients (r) among the average gene expression profiles of the 11 identified cell clusters were generated on the basis of the top 2,000 highly expressed genes. The color scale ranged from blue (low correlation) to red (high correlation), with the expression levels indicated in each cell.


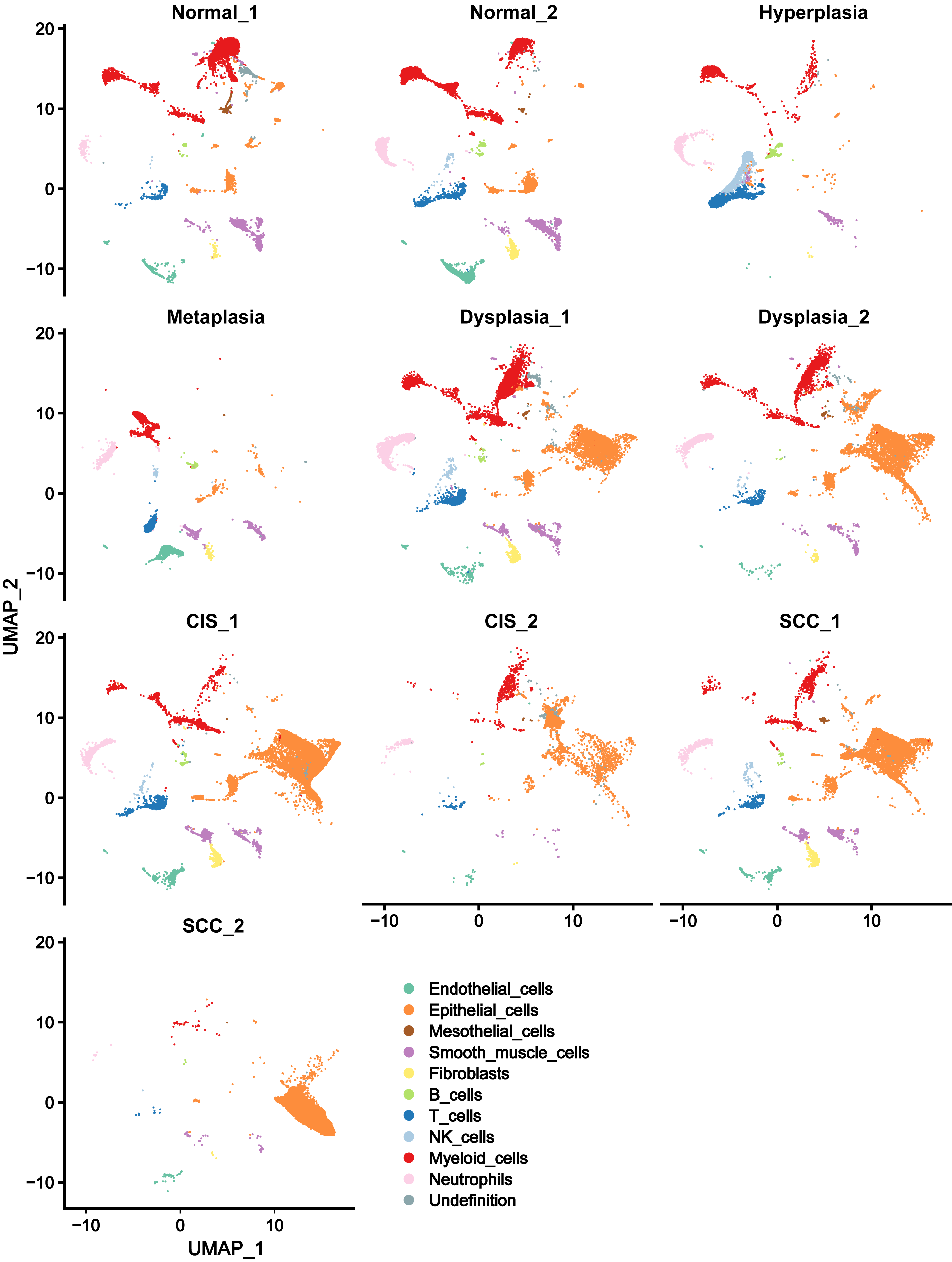


**Supplementary Figure 5 | UMAP** **projection of the cell distribution**. Distribution of 11 distinct cell subclusters at different lesion stages. Each point represents a single cell. The x- and y-axes represent t-SNE dimensions 1 and 2, respectively.


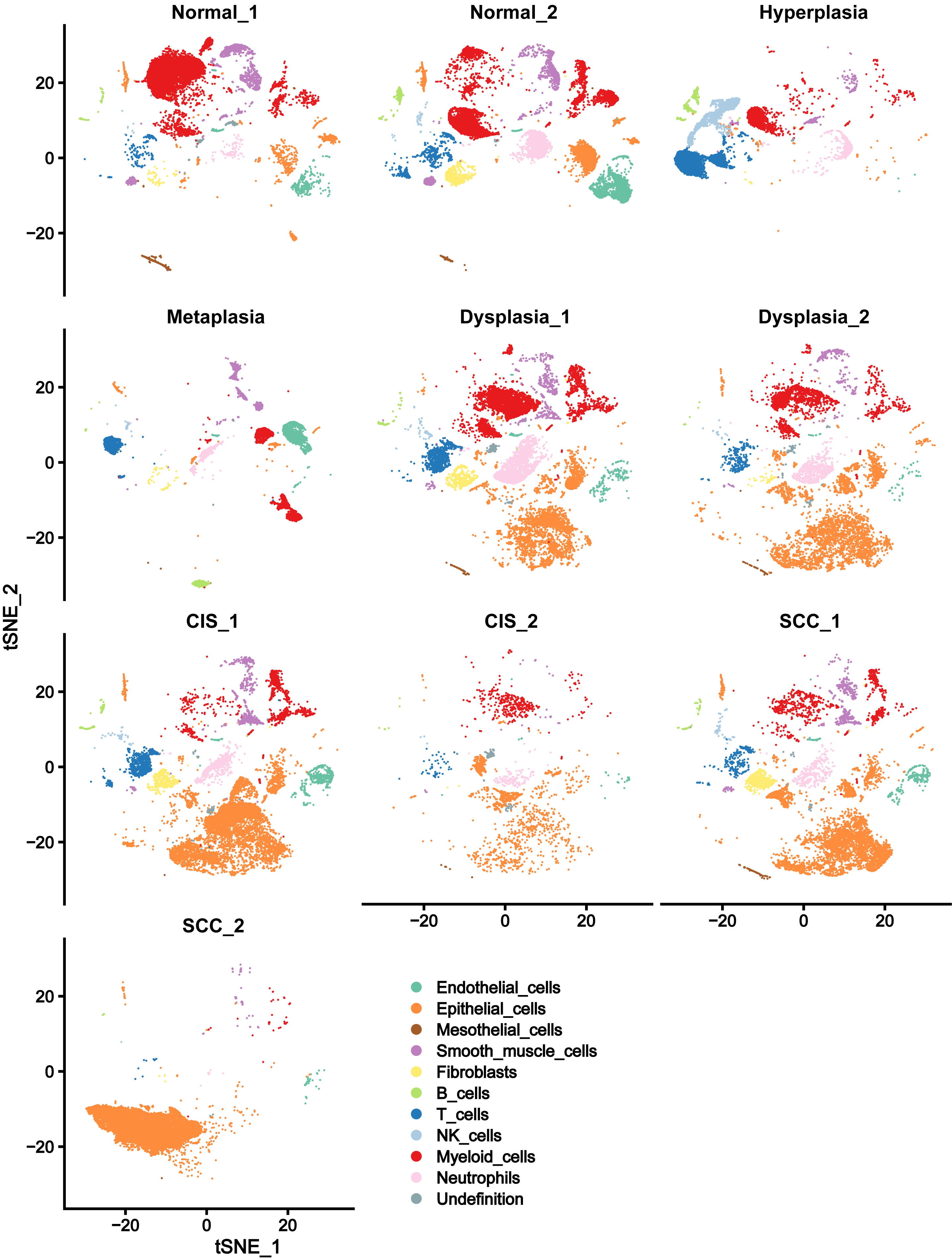


**Supplementary Figure 6 | t-SNE projection of the cell distribution.** Distribution of 11 distinct cell subclusters at different lesion stages. Each point represents a single cell. The x- and y-axes represent t-SNE dimensions 1 and 2, respectively. This t-SNE visualization serves as a supplementary explanation to the UMAP analysis in Supplementary Figure 5, providing an alternative perspective on the cellular landscape.


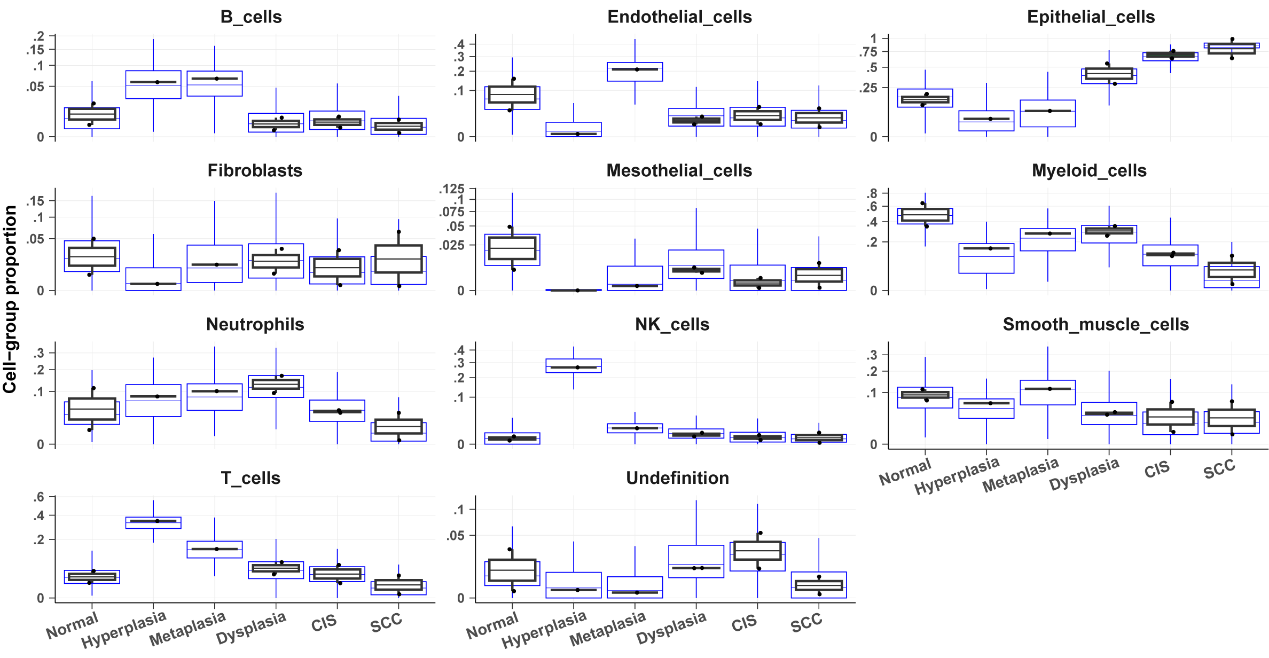


**Supplementary Figure 7 | Proportion distributions of the cell types using sccomp.** The blue box plots represent the posterior predictive check.


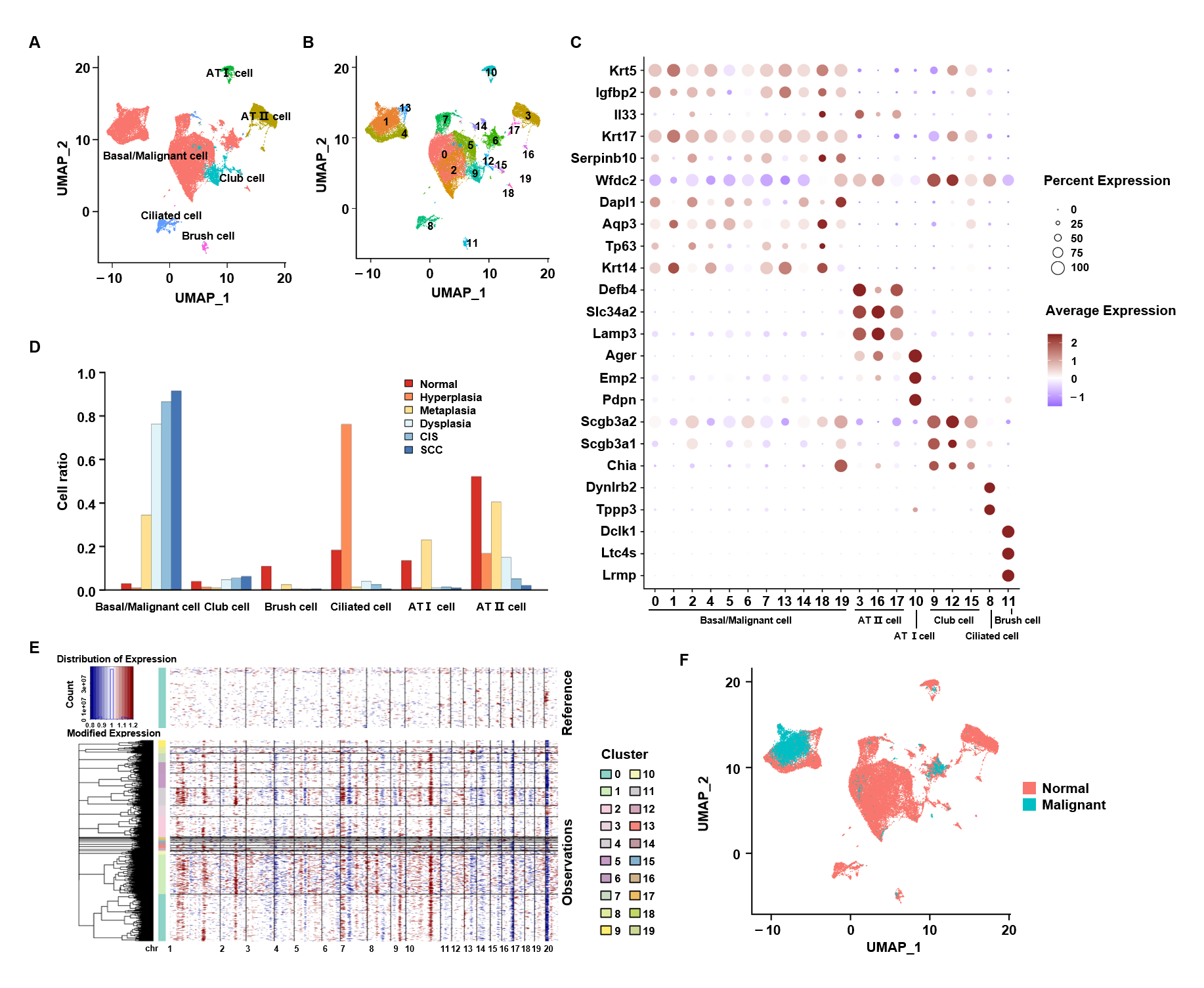


**Supplementary Figure 8 | Subcluster diversity and CNV analysis of epithelial cells.** **A-B,** UMAP plot showing different epithelial cell subclusters from 10 samples color-coded by major cell types (**A**) and subclusters (**B**). **C,** The average expression levels of signature genes specific for each of the 20 subclusters depicted in (**B**). **D,** Relative ratio of each cell type across the six lesion stages. **E,** Merged hierarchical heatmap of CNVs for epithelial cells from all samples. **F,** UPAP plot showing malignant and normal cells, inferred from the CNV analysis.


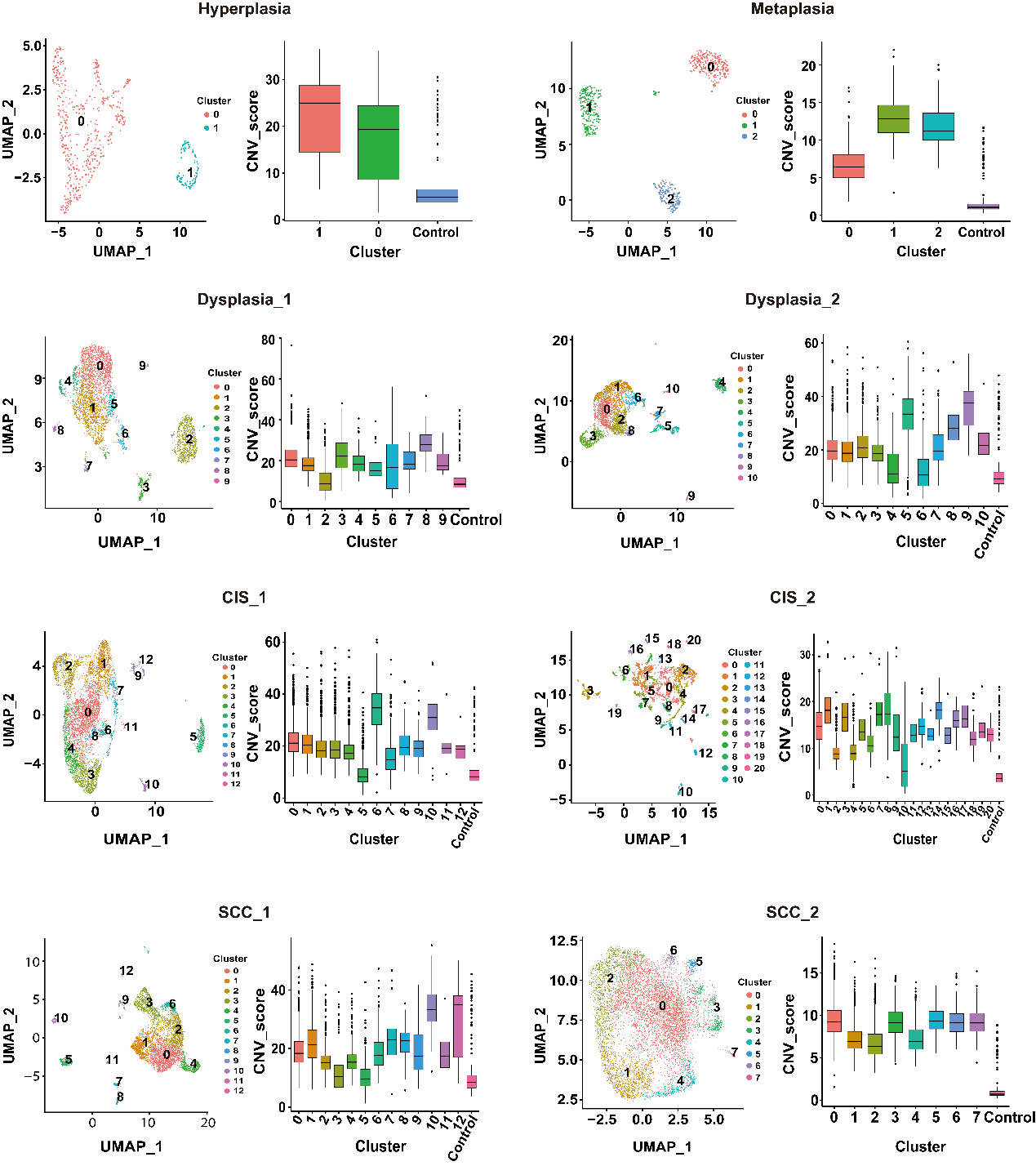


**Supplementary Figure 9 | Epithelial cell subclusters of each sample and corresponding CNV scores.**


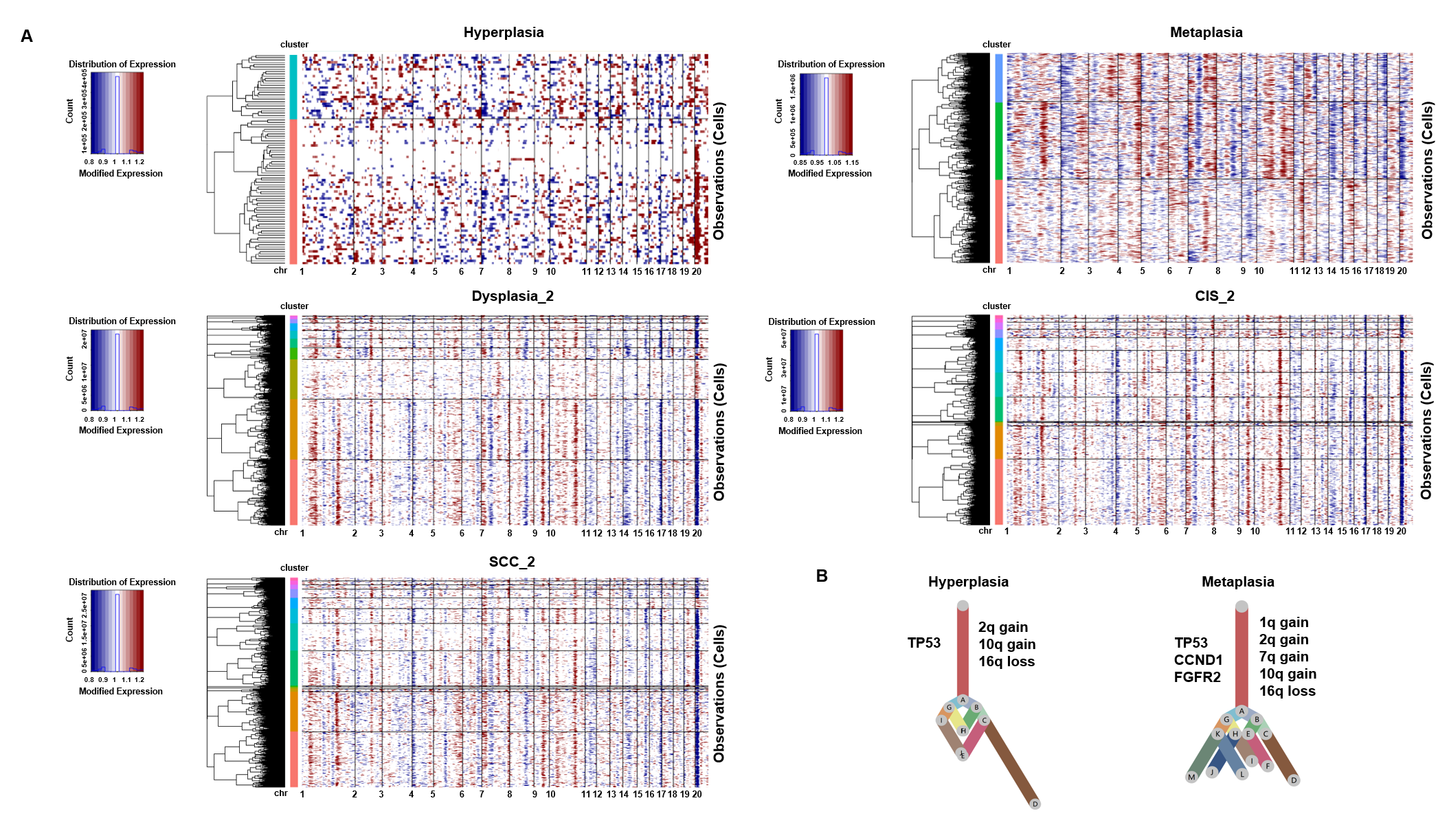


**Supplementary Figure 10 | Single-cell CNV analysis.** **A,** Hierarchical heatmaps of CNVs from the inferCNV algorithm in hyperplasia, metaplasia, dysplasia, CIS, and SCC lesions. The annotation track on the left indicates the epithelial cell subclusters depicted in Supplementary Figure 9. The red blocks and blue blocks represent copy number gains and copy number losses, representatively. **B,** Clone evolutionary trees of hyperplasia and metaplasia lesions generated via uphyloplot2 mapping on the basis of CNVs and relevant key genes.


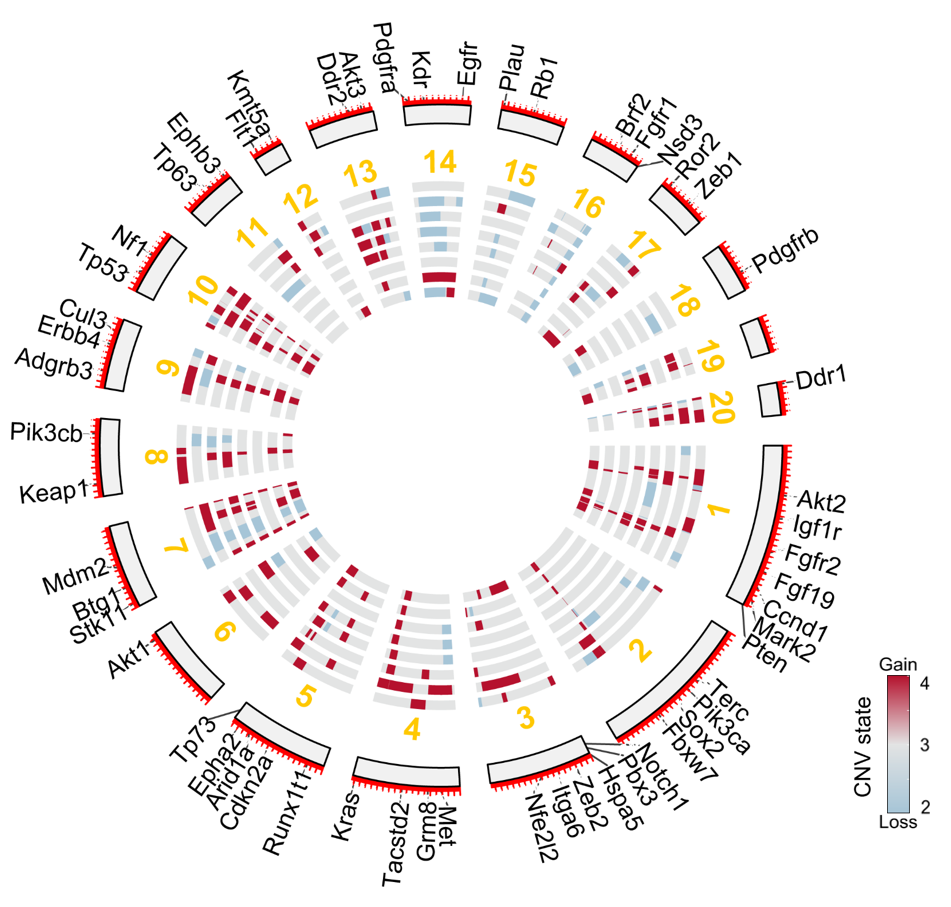


**Supplementary Figure 11 | Genomic aberrations in different stages of LUSC development.** In the center of the Circos plot, eight concentric rings represented the CNV profiles of eight samples spanning hyperplasia, metaplasia, dysplasia, CIS, and SCC lesions. The outermost ring in the Circos plot denotes the chromosome coding. Genes previously identified as potential drivers of LUSC are labeled. The color scale ranged from red (copy number gains) to blue (copy number losses).


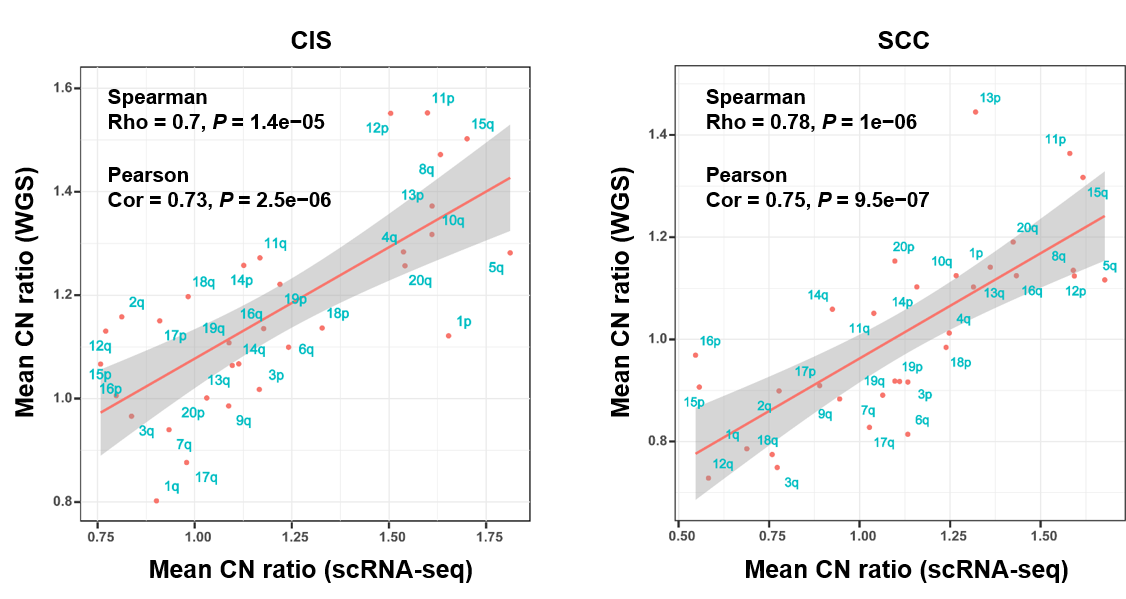


**Supplementary Figure 12 | Correlation analysis of the large-scale CNVs inferred from scRNA-seq and WGS**. Pearson and Spearman correlations were computed on arm-level copy number (CN) ratios from both datatypes. Representative CIS and SCC samples are shown.


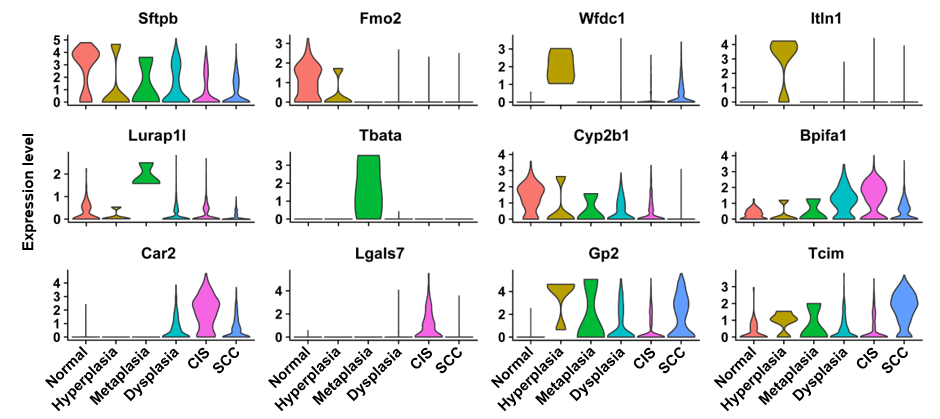


**Supplementary Figure 13 | Expression levels of differentially expressed genes in club cells at various stages.**


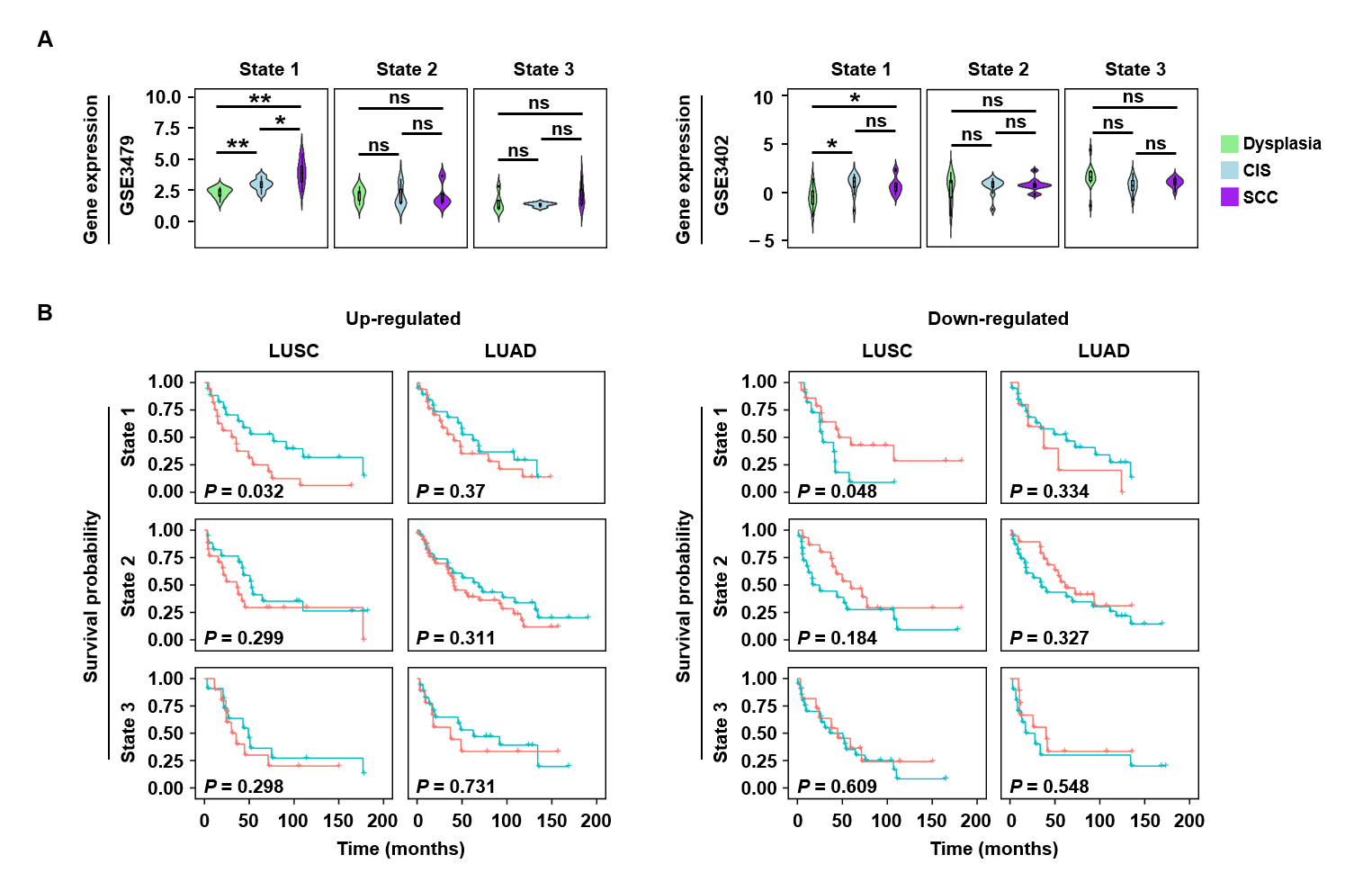


**Supplementary Figure 14 | Validation of three state-specific signatures in human cohorts. A,** Analysis of the average expression of the top 10 state-specific signature genes were validated in two independent GEO datasets (GSE33479, n = 40 samples; GSE73402, n = 62 samples). **B,** Kaplan–Meier OS curves from institutional cohort, stratified by the mean expression of up-regulated genes (left) and down-regulated genes (right). **p* < 0.05, ***p* < 0.01; ns, not significant; two-sided Wilcoxon test (**A**) and two-sided log-rank test (**B**) were used.


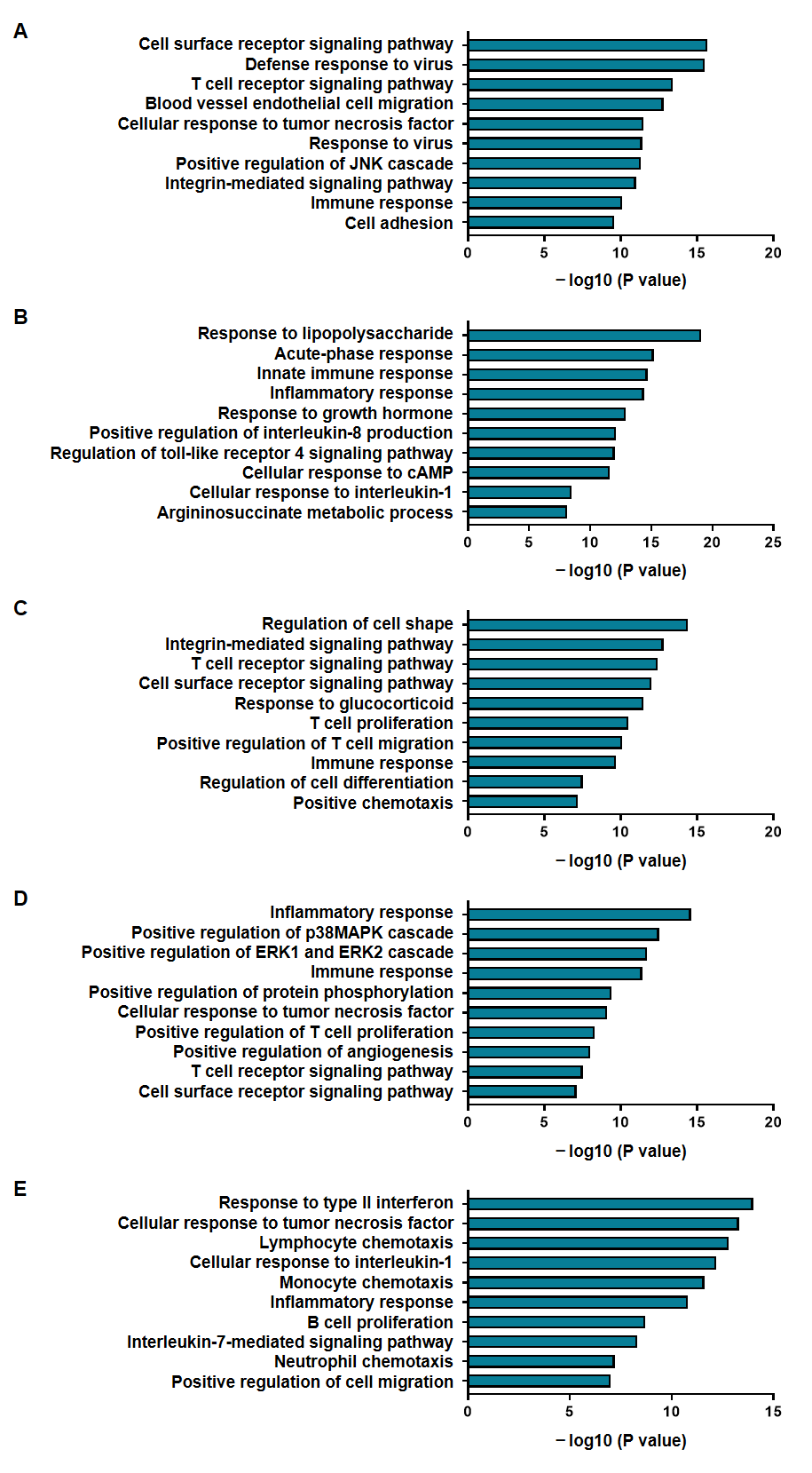


**Supplementary Figure 15 | GO enrichment analysis of monocyte clusters**. **A,** Selected GO enrichment of classical monocytes (Cluster 4). **B,** Selected GO enrichment of nonclassical monocytes (Cluster 2). **C,** Selected GO enrichment of nonclassical monocytes (Cluster 5). **D,** Selected GO enrichment of nonclassical monocytes (Cluster 8). **E,** Selected GO enrichment of nonclassical monocytes (Cluster 15). The *x*-axis indicates the -log10-transformed false-discovery rate (FDR)-adjusted *p* values.


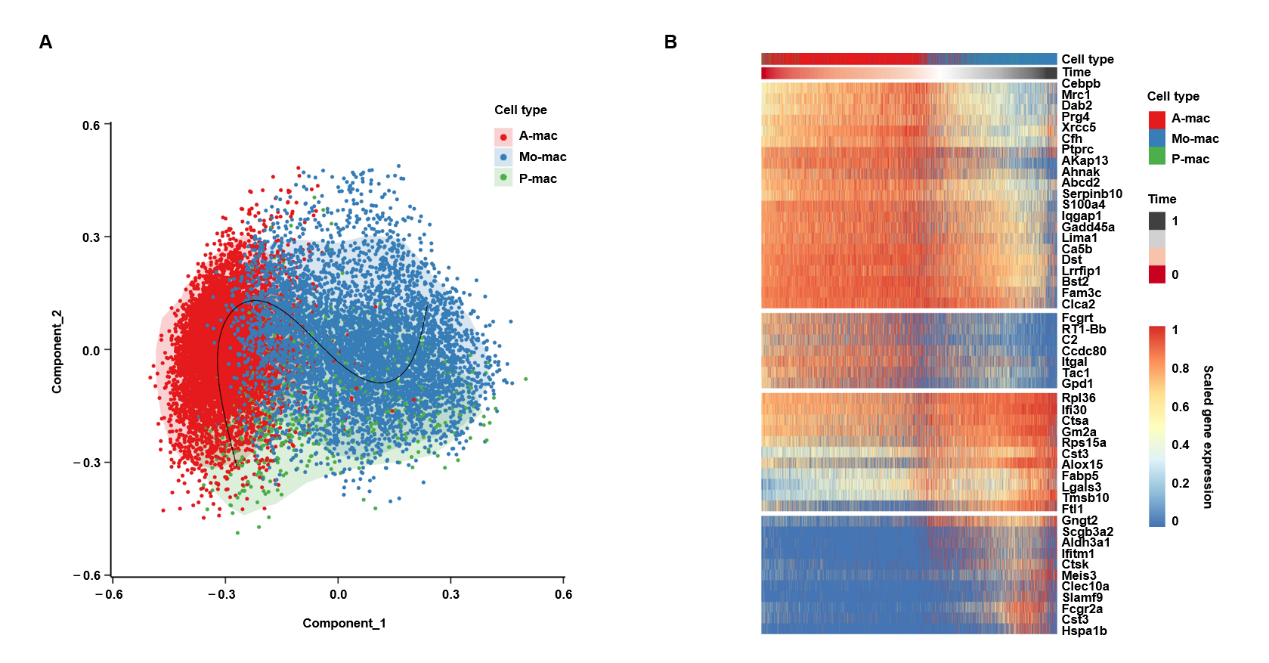


**Supplementary Figure 16 | Trajectory analysis of macrophage differentiation using the SCORPIUS algorithm.** **A,** Trajectory plot of macrophage differentiation inferred by the SCORPIUS algorithm. The cell types defined by the UMAP analysis were color-coded. **B,** Heatmap of the gene expression profiles of the top 50 differently expressed genes along the pseudotime trajectory as indicated. These genes were grouped into four clusters based on their expression pattern. The color key from blue to red indicates the scaled gene expression levels in macrophages.


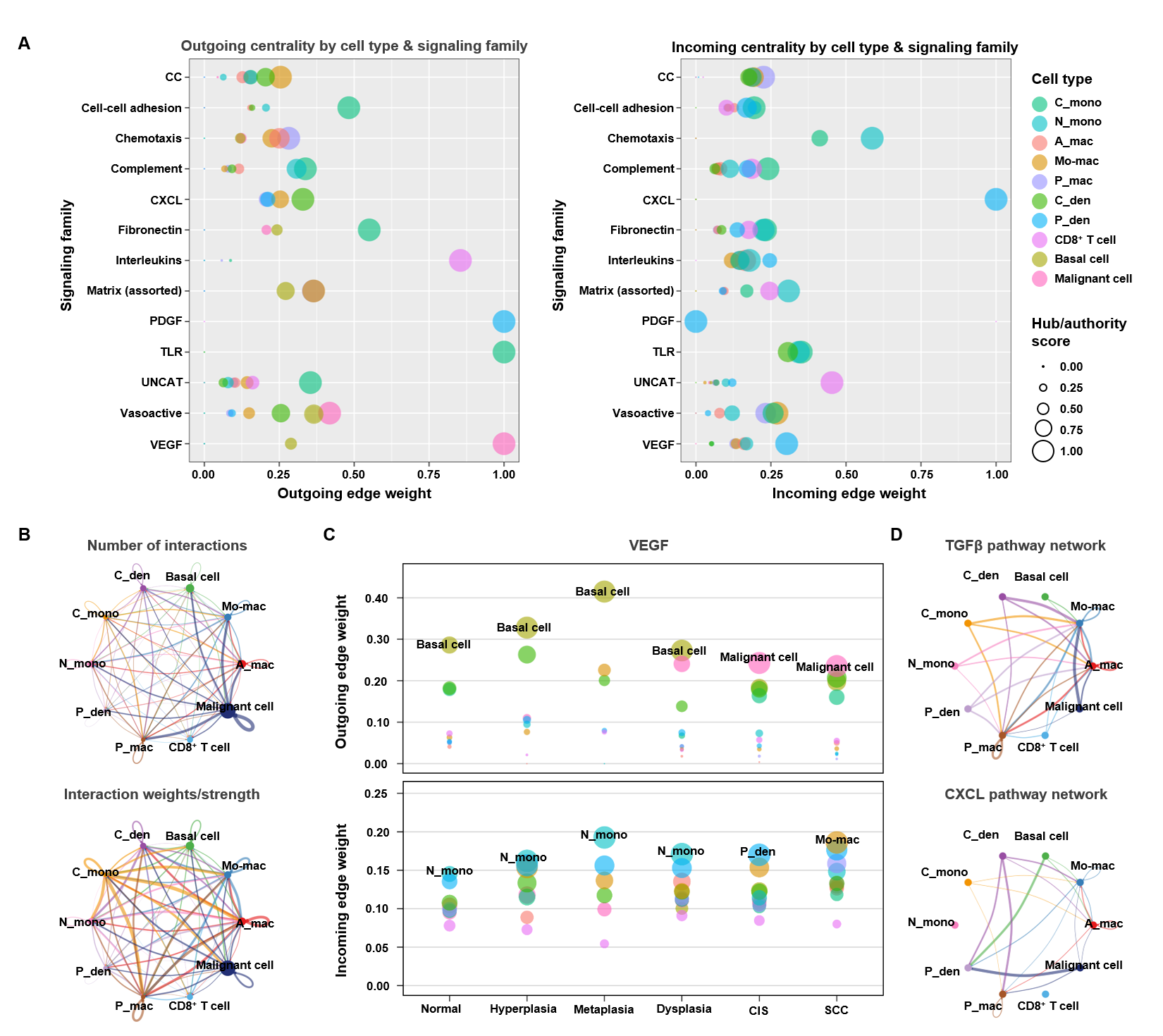


**Supplementary Figure 17 | Cell–cell interaction networks revealed by Connectome. A,** Identification of signaling family networks for various cell types. The left pane displays outgoing centrality (cumulative outgoing edge weight and Kleinberg hub scores) while the right panel shows incoming centrality metrics (cumulative incoming edge weight and Kleinberg authority scores). **B,** The number and the strength of interaction between populations. **C,** Centrality analysis at each stage for all VEGF-mediated edges. Basal cells and malignant cells occupy a dominant position in information transmission. Recruited plasmacytoid dendritic cells and monocyte-derived macrophages take over nonclassical monocytes with lesion progression. **D,** Cell-cell communication involving TGFβ and CXCL signaling pathways.
